# Armoring Black Phosphorus Anode with Stable Metal–Organic-Framework Layer for Hybrid K-Ion Capacitors

**DOI:** 10.1007/s40820-020-00570-7

**Published:** 2021-01-04

**Authors:** Mengzhu Xu, Yutong Feng, Bingjie Chen, Ruijin Meng, Mengting Xia, Feng Gu, Donglei Yang, Chi Zhang, Jinhu Yang

**Affiliations:** 1 Research Center for Translational Medicine & Key Laboratory of Arrhythmias of the Ministry of Education of China, East Hospital, Tongji University School of Medicine, Shanghai, 200120 People’s Republic of China; 2 School of Chemical Science and Engineering, Tongji University, Shanghai, 200092 People’s Republic of China; 3 Institute for Process Modelling and Optimization, Jiangsu Industrial Technology Research Institute, SIP, 388 Ruoshui Road, Suzhou, Jiangsu People’s Republic of China; 4 Institute of Molecular Medicine School of Medicine, Shanghai Jiao Tong University, Shanghai, 200127 People’s Republic of China

**Keywords:** Black phosphorus, MOF, Interphase engineering, Potassium-ion capacitor, Cycling stability

## Abstract

Potassium-ion capacitors (KICs) are promising for sustainable and eco-friendly energy storage technologies, yet their slow reaction kinetics and poor cyclability induced by large *K*-ion size are a major obstacle toward practical applications. Herein, by employing black phosphorus nanosheets (BPNSs) as a typical high-capacity anode material, we report that BPNS anodes armored with an ultrathin oriented-grown metal–organic-framework (MOF) interphase layer (BPNS@MOF) exhibit regulated potassium storage behavior for high-performance KICs. The MOF interphase layers as protective layer with ordered pores and high chemical/mechanical stability facilitate *K* ion diffusion and accommodate the volume change of electrode, beneficial for improved reaction kinetics and enhanced cyclability, as evidenced by substantial characterizations, kinetics analysis and DFT calculations. Consequently, the BPNS@MOF electrode as KIC anodes exhibits outstanding cycle performance outperforming most of the reported state-of-art KICs so far.

**Electronic supplementary material:**

The online version contains supplementary material available at (10.1007/s40820-020-00570-7) contains supplementary material, which is available to authorized users.

## Highlights

- The ultrathin metal–organic-framework (MOF) interphase layer with high mechanical/chemical stability was in situ grown on black phosphorus nanosheets (BPNSs).
- MOF interphase layers as an ordered porous and robust protective layer can facilitate K ion diffusion and accommodate the volume change of the electrode.
- Benefiting from the improved reaction kinetics and enhanced electrode stability, the BPNS@MOF anode for potassium-ion capacitors exhibits outstanding cycle performance.

**Electronic supplementary material:**

The online version contains supplementary material available at (10.1007/s40820-020-00570-7) contains supplementary material, which is available to authorized users.

## Graphical abstract

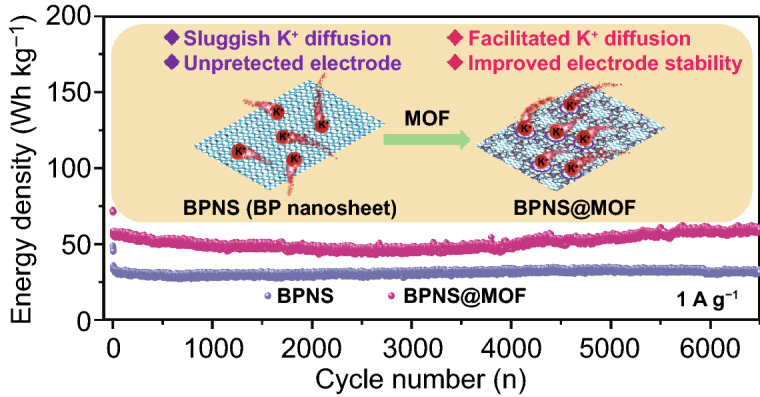

## Introduction

Recently, potassium-based electrochemical energy storage devices have attracted ever-increasing attention due to the natural abundance of potassium element and the low redox potential of K/K^+^ (− 2.92 V vs SHE) close to that of Li/Li^+^ (− 3.04 V vs SHE) [1–3]. So far, potassium (*K*)-ion batteries (KIBs) are the most attracting potassium-based electrochemical energy storage device and extensive research efforts have been carried out [4, 5]. However, similar to other alkali metal ion batteries, KIBs suffer from inferior cycling lifespan and insufficient power density [6]. On the other hand, hybrid potassium (*K*)-ion capacitors (KICs) have gained particular interest and are expected as the replacement for KIBs due to their unique characteristic that couples a capacitor-type cathode with a battery-type anode, which are expected to deliver outstanding energy at high power [7–15]. Thereinto, battery-type anodes associating with complicated potassiation/depotassiation behaviors are considered as the key component affecting the performance of KICs. To date, various candidate materials, such as carbon-based materials [7, 8], V_2_C MXene [9], Ca_0.5_Ti_2_(PO_4_)_3_@C [10], K_2_Ti_6_O_13_ [11], organic K_2_TP [12], Co_2_P [13], N-MoSe_2_/G [14] and MoS_2-x_Se_x_/C-HNTs [15], have been investigated as anodes for KICs. Despite much progress, the comprehensive performance of KICs based on these anode materials is still far from satisfying the requirements. Therefore, the exploration of new anode materials with regulated potassium storage behaviors is highly desired for the development of high-performance KICs.

Black phosphorus (BP) has been recognized as a superior electrode material for electrochemical energy storage systems, including organic and aqueous alkaline metal ion batteries [16, 17], Li–S batteries [18] and hybrid ion capacitors [19], due to its unique properties such as high carrier mobility and high energy storage capacity [20, 21]. As an alloy-based anode material, BP offers superior specific capacity (1154 mAh g^−1^ for K_4_P_3_) [22, 23] compared to the traditional K-ion intercalation materials [24–26]. However, BP-based materials have not yet been explored in KICs. The most serious challenge is the dramatic volume expansion causing electrode pulverization induced by the large ion size of K^+^ (1.38 Å vs. 0.76 Å of Li^+^) [27]. In most cases, carbon-based materials were employed as the protective layers or matrix for anode materials to accommodate the volume expansion and keep the integrity of the electrodes. However, this strategy cannot be applicable to BP which possesses a low sublimation temperature (530 °C) and is sensitive to water and oxygen, because the introduction of carbon materials was usually realized through high-temperature carbonization or hydrothermal procedures.

Zeolitic imidazolate frameworks (ZIFs) are a subclass of metal–organic frameworks (MOFs) and possess similar structures to zeolites. Among which, ZIF-8 is constructed from tetrahedrally coordinated zinc ions linked by organic dimethyl imidazole (Mim) units [28]. ZIF-8 has attracted significant interest for its easy preparation, ordered nanoporous structure, exceptional mechanical stability and ultrahigh chemical stability resulting from the metal-nitrogen bonds [29]. Notably, it was reported that ZIF-8 could withstand metal ion intercalation/deintercalation [30–32], preserving its structure/chemical composition intact during cycles. For example, Fan et al. introduced a stable artificial solid electrolyte interphase (SEI) film prepared by polyvinyl alcohol (PVA) cementing a metal–organic framework (Zn-MOF), which is beneficial for inhibiting dendrite growth and easing the volume change [30]. Li et al. reported the commercial LiNi_1/3_Co_1/3_Mn_1/3_O_2_ (NCM_333_) cathode modified by synthesizing ZIF-8 in situ on the surface of NCM_333_, in which ZIF-8 can not only act as a shield against the electrolyte corrosion, but also enable faster lithium-ion transfer at the interfacial regions [31]. Liu et al. proposed an in situ grown porous ZIF-8 as an ideal Zn^2+^ modulation layer to effectively regulate the aqueous Zn deposition behavior [32]. The unique properties make ZIF-8 a promising protective layer for BP.

In this work, ultrathin MOF interphase (MI) layer made of ZIF-8 has been constructed on the surface of black phosphorus nanosheets (BPNSs) by a in situ oriented-grown strategy and the fabricated composite material (denoted as BPNS@MOF) is employed as representative anodes for KICs. The as-prepared BPNS@MOF exhibits a set of merits as anodes for KICs: (i) ultrathin MI layers as protective layer with ordered nanopores are beneficial to efficient *K* ion transport; (ii) the high chemical and structural stability of ZIF-8 plus the robust interface can effectively accommodate the volume change of BP to prevent electrode pulverization; (iii) the thin thickness of the BPNSs can both shorten the diffusion length of *K* ions and effectively reduce the large volume change of the electrode during charge/discharge processes. Accordingly, the hybrid KIC constructed by coupling the BPNS@MOF anode and activated carbon (AC) cathode can deliver a significantly improved energy density compared with the BPNS-based KIC and, particularly, outstanding cycle performance outperforming most of the reported state-of-art KICs so far.

## Experimental Section

### Synthesis of the BPNSs

Prior to the synthesis, bulk BP crystals were firstly prepared according to the previously reported method [33] by using red phosphorus, tin and iodine as source materials sealed in a vacuumed ampule in a muffle furnace. The black phosphorus nanosheets were prepared by electrochemical cationic intercalation [34] and liquid exfoliation of the prepared bulk BP crystals. In a typical experiment, BP crystals (8 mm in length, 5 mm in width) were used as the cathode and a Pt foil (10 mm in length, 10 mm in width) was employed as the counter electrode. The distance between the counter electrode and the working electrode was kept at ≈ 1.5 cm. Static potentials of − 5 V were applied to the bulk BP electrodes using an electrochemical workstation (CHI 660e). The electrolyte was prepared by dissolving TBAB (16.1 g) in DMF (100 mL), and the exfoliation was lasted for 1 h. Then, the materials were sonicated in an ice bath for 2 h at a power of 400 W. Subsequently, the dispersions were separately washed with DMF and methyl 10 times to remove TBAB and DMF in the solution. Next, the materials were dispersed in methyl and the resultant brown suspension was centrifuged at 1000 rpm for 10 min to remove the residual unexfoliated bulk BP. Afterward, the resultant BPNS dispersions were centrifuged for 10 min at 10,000 rpm to collect the products, followed by drying in a vacuum oven at 60 °C for 12 h.

### Synthesis of the BPNS@MOF

In a typical procedure, 50 mg of as-prepared few-layer BPNSs were dispersed into 150 mL of methanol containing 1.65 g of PVP (K29-32). After ultrasonic dispersion, 0.0745 g of Zn(NO_3_)_2_·6H_2_O (0.25 mmol) was subsequently dissolved into the mixed solution and stirred for 2 h at room temperature. Then, 0.137 g (1.67 mmol) of 2-methylimidazole was added into the above solution (the molar ratio of Zn(NO_3_)_2_·6H_2_O:2-Mim was 1:6.68), followed by vigorous stirring for 4 h at room temperature. Finally, the product was collected by centrifugation and washed with methanol for three times, followed by drying in a vacuum oven at 60 °C overnight and then stored in a glove box filled with argon before use. In the typical synthesis, the product is denoted as BPNS@MOF. For comparison, BPNS@MOF-2, BPNS@MOF-3 were also prepared through the same approach adding of 0.00298 and 0.149 g Zn(NO_3_)_2_·6H_2_O, respectively, while the ratio of Zn(NO_3_)_2_·6H_2_O/2-Mim remains unchanged.

### Material Characterization

The morphologies of the as-prepared samples were observed using a scanning electron microscopy (SEM, Hitachi S-4800, 3 kV) and transmission electron microscope (TEM, Talos F200X G2) together with associated energy-dispersive X-ray spectroscopy (EDS). Atomic force microscopy (Dimension ICON with NanoScope V controller, Bruker, USA) was used to characterize the samples in tapping mode in air. Samples were dropped onto a thermally oxidized Si (300 nm SiO_2_) substrate for AFM measurement. X-ray diffraction patterns (XRD) were recorded on a D8 advance X-ray diffractometer with a Cu Kα radiation source (λ = 0.15418 nm) in a step of 0.02° over a 2θ range of 5–70°. TriStarII 3020 system is used to characterize nitrogen adsorption–desorption isotherms, the sample was degassed in vacuum at 200 °C for at least 4 h. The Brunauer–Emmett–Teller (BET) method was utilized to calculate the surface areas. The pore size distributions were retrieved by using the Barrett-Joyner-Halanda (BJH) method from the adsorption branch of the isotherms. X-ray photoelectron spectroscopy (XPS) investigation was conducted on PHI-5000C ESCA system (Perkin Elmer) with Mg Kα radiation, and the binding energies (BEs) were referred of the C 1 s peak at 284.80 eV. Raman spectra were measured by using a spectrophotometer (Invia, Renishaw, Germany) with a 514 nm laser. Fourier transform infrared spectra were recorded on Nicolet 6700 FT-IR spectrometric analyzer.

### Electrochemical Measurements

For fabrication of the anode electrodes, the active material (BPNSs or BPNS@MOF), carbon black additive and poly(acrylic acid) (PAA) binder were prepared into a slurry in N-methyl-2-pyrrolidone (NMP) with a weight ratio of 7:2:1, followed by pasting the slurry onto a copper foil current collector. The areal mass loading of the anode is about 1.2–1.5 mg cm^− 2^. For fabrication of the cathode electrodes, the active carbon (AC), polyvinylidene fluoride (PVDF) binder and carbon black additive were mixed with a weight ratio of 8:1:1, followed by pasting the slurry onto an aluminum foil current collector. The electrodes were dried at 80 °C for 24 h under vacuum before the assemble of cells. Both hybrid KICs and half-cells of the KIBs were assembled inside an Ar filled glovebox where the oxygen and water concentrations are kept below 0.01 ppm. Hybrid KICs were assembled with CR2032 coin cells, employing a glass fiber filter (Whatman GF/D) as the separator, 1.0 mol L^−1^ KPF_6_ in ethylene carbonate:diethyl carbonate (1:1, volume ratio) as the electrolyte. Before assembling the devices, the BPNS@MOF (or BPNS) anode electrodes were preactivated in half-cells by charging/discharging at 50 mA g^−1^ for 5 cycles. Then, the cells were disassembled inside the glove box and a KIC was assembled employing the preactivated anode and activated carbon cathode. The anode to cathode mass ratio was 4:1. For comparison, KICs with different anode to cathode mass ratio of 3:1, 5:1 were also prepared. Half-cells of the KIBs were assembled with CR2016 coin cells, employing a glass fiber filter (Whatman GF/F) as the separator, 1.0 mol L^−1^ KFSI in ethylene carbonate:diethyl carbonate (1:1, volume ratio) as the electrolyte, *K* metal as the counter electrode. All the electrochemical tests were carried out at room temperature. Galvanostatic discharge–charge cycling was performed using a Land-2001A (Wuhan, China) automatic battery tester. Cyclic voltammetry was performed on an electrochemical workstation (CHI 660e) at a scan rate of 0.01 mV s^−1^, and the electrochemical impedance spectra were recorded using the same electrochemical workstation by applying an AC voltage of 5 mV amplitude over the frequency range from 100 kHz to 0.01 Hz. The gravimetric energy and the power densities of the KIC device were calculated by numerically integrating the galvanostatic discharge profiles using the equations as follows:

1 $$E_{mass} = \int_{t2}^{t1} IU/mdt$$

2 $$P_{mass} \, = \,E_{mass} /t$$

where *I* is the constant current (*A*), *U* is the working voltage (*V*), *m* is the total mass of the electrode, *t*_*1*_ and *t*_*2*_ are the start/end-of-discharge time (*s*) of the KIC device, respectively, and *t* is the discharge time (*s*).

### Theoretical Calculation

DFT calculations were performed using the Vienna ab initio simulation package. The exchange–correlation energy was described by the generalized gradient approximation (GGA) proposed by Perdew, Burke and Ernzerhof (PBE) [35]. The electronic wave functions were expanded using a plane wave basis set with a kinetic energy cutoff of 400 eV with a 2 × 2 × 1 Monkhorst–Pack sampled k points. The model was built by using the (020) plane of black phosphorus, the (112) plane of ZIF-8 and the (002) plane of K_2_SO_4_. To find out the minimum energy path (MEP) and saddle points between the initial and final positions and calculate the migration energy barrier for K diffusion, the climbing image nudged elastic band (CI-NEB) method was employed [36].

## Results and Discussion

### Synthesis and Characterizations of the BPNS@MOF

**Fig. 1:**
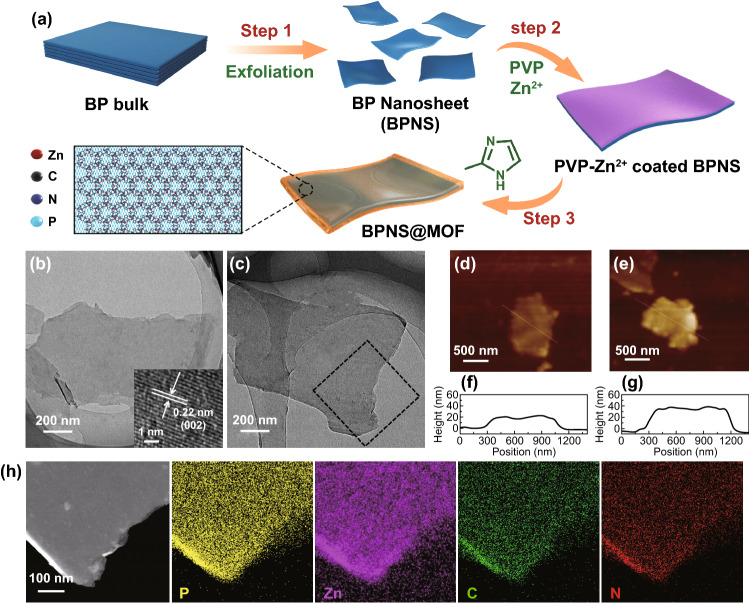
**a** Schematic illustration of the formation process of the BPNS@MOF. **b, c** TEM images of the pristine BPNSs (**b**) and BPNS@MOF (**c**). The inset in (**b**) is a HRTEM image showing crystal lattices for the BPNSs. The black dotted box in (**c**) corresponds to the area of element mappings in (**h**). **d, e** AFM images of BPNSs (**d**) and BPNS@MOF (**e**). **f, g** Height profiles along the white lines in (**d**) and (**e**). **h** Scanning TEM image and the corresponding element mappings of P, Zn, C and N elements of the BPNS@MOF

The overall synthesis process for the BPNS@MOF samples is presented in Fig. 1a. Briefly, the BPNSs were obtained through the exfoliation of bulk BP by electrochemical cationic intercalation [34] (Step 1). Subsequently, the as-exfoliated BPNSs were coated with a thin layer of polyvinylpyrrolidone (PVP) and abundant Zn ions on the surface through the electrostatic interaction in the presence of PVP and Zn(NO_3_)_2_ in methanol solution, consecutively (Step 2). Then, in situ growth of ZIF-8 layer on surface of the BPNSs was triggered by the addition of the ligand of 2-methylimidazolate [37], forming a crystalline MI layer with ordered pores on the BPNSs as well as the final product of the BPNS@MOF (Step 3).

**Fig. 2:**
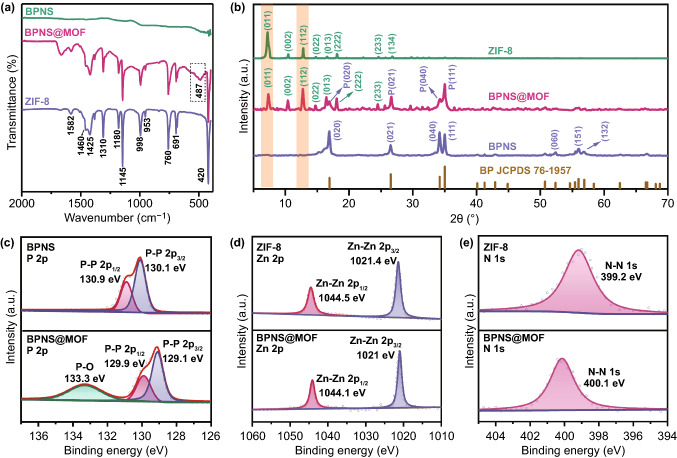
**a** FTIR spectra of the BPNSs, BPNS@MOF and ZIF-8. **b** XRD patterns of the BPNSs, BPNS@MOF and ZIF-8. **c** High-resolution P 2p XPS spectra of the BPNSs and BPNS@MOF. **d** Zn 2p and **e** N 1 s XPS spectra of ZIF-8 and BPNS@MOF

Figure 1b, c shows TEM images of the as-exfoliated BPNSs and the typical BPNS@MOF, respectively. The BPNSs display a typical nanosheet-like shape and smooth surface, with the diameter up to several micrometer (Figs. 1b and S1a, b). A high-resolution TEM (HRTEM) image (inset in Fig. 1b) displays clear lattice fringes with d-spacings of ≈0.22 nm, corresponding to the (002) plane of BP (JCPDS No. 76–1957) [38], which also confirms the top face of the BPNSs is bounded by the {020} planes. The BPNS@MOF holds a similar nanosheet-like morphology to the pristine BPNSs (Figs. 1c and S1c, d) and a much thicker thickness (~ 34 nm) relative to the BPNSs (~ 20 nm) revealed by the corresponding atomic force microscopy (AFM) line scan image (Fig. 1d-g), indicating the thickness of the MI layer in the BPNS@MOF is about 7 nm. In addition, the corresponding element mappings in Fig. 2h (the area enclosed by the dotted line in Fig. 2c) demonstrate that P, Zn, C and N elements are uniformly distributed throughout the whole nanosheet, indicating the successful growth of a MI layer on the BPNSs with high uniformity. Although the MOF layer definitely exists and uniformly distribute on BPNSs as evidenced by AFM and element mapping, it cannot be directly observed by TEM at a high magnification (Fig. S2) as the MOF is crystalline. The mass percentage of MOF and BP in the typical BPNS@MOF sample was measured to be 45.51 and 54.49%, respectively, by inductive coupled plasma emission spectrometer (ICP-MS). For comparison, BPNS@MOF samples with different MOF contents, i.e., a lower content of 25.05% and a higher content of 56.11% were also prepared and denoted as BPNS@MOF-2 and BPNS@MOF-3, respectively (Figs. S3a–d and S4). The BPNS@MOF-2 and BPNS@MOF-3 also show nanosheet-like morphology similarly to the BPNSs and BPNS@MOF, yet distinct from ZIF-8 polyhedral nanocrystals synthesized in the absence of the BPNSs (Fig. S3e, f). It is found that for the BPNS@MOF-3, obvious ZIF-8 nanoparticles with sizes of 20–50 nm appear on BPNSs, implying the self-nucleation of ZIF-8 was occurred due to the high MOF content.

Fourier transform infrared spectrometer (FTIR) spectra in Fig. 2a prove the successful preparation of the BPNS@MOF. For the BPNSs, no apparent peak is observed in the FTIR spectrum, indicating that the BP is infrared inactive. For the BPNS@MOF, all peaks match well with ZIF-8 derived from various vibration modes [39]. Specifically, the intense vibrational bands at 1350–1500 cm^−1^ are associated with the entire ring stretching vibrations of the imidazole moiety and the bands in the region of 900–1350 cm^−1^ are ascribed to the in-plane bending of the imidazole ring. The peak at 420 cm^–1^ can be assigned as the Zn–N stretch mode [39], which confirms the coordination between metal clusters and organic linkers in the framework. The peak at 487 cm^–1^ can be ascribed to the Zn–P bond vibrations [40].

It should be noted that no peak corresponds to PVP in the FTIR spectrum of the BPNS@MOF, proving that PVP was completely removed after washing process. The N_2_ adsorption–desorption isotherms in Fig. S5a show the specific surface area (S_BET_) of the BPNS@MOF is ~ 278.8 m^2^ g^−1^ with pore sizes in the range of 1–3 nm. Obviously, the S_BET_ value was significantly increased after coating MI layer on the BPNSs (S_BET_ = 6.1 m^2^ g^−1^).

Figure 2b shows the XRD patterns of the BPNSs, BPNS@MOF as well as ZIF-8 crystals. The XRD pattern of the BPNS@MOF exhibits a set of diffraction peaks assigned to orthorhombic BP (JCPDS No. 76–1957) [38] and cubic ZIF-8 [41], respectively. The results indicate the MI layers are crystalline and also confirm the formation of the BPNS@MOF. Interestingly, it is noted that the peak corresponding to the (011) plane is the strongest in pristine ZIF-8 crystal while the (112) peak become the strongest in the BPNS@MOF, suggesting that the (112) plane becomes dominant in the MI layer and an oriented growth of (112) plane occurs during deposition of the MI layer. To probe the reason of the change of dominant plane, XPS examination was conducted for the BPNS@MOF, BPNSs and ZIF-8 (Figs. 2c-e and S5b–d). A survey XPS spectrum of the BPNS@MOF evidences the presence of P, Zn, C, N and O in the material (Fig. S5b). The high-resolution P 2p spectrum of the BPNS@MOF displays two peaks at 129.1 and 129.9 eV [42], showing a reduction of 1 eV in binding energy compared with the BPNSs (Fig. 2c). In addition, the peaks for Zn 2p at 1021.0 and 1044.1 eV also show a negative shift (− 0.4 eV) [43] compared with the ZIF-8 (Fig. 2d). While N 1 s of the BPNS@MOF exhibits a single peak at 400.1 eV [44], which has a binding energy increase of 1 eV relative to that of ZIF-8. Accordingly, the binding energy decrease of P 2p, Zn 2p and the increase of N 1 s for the BPNS@MOF is considered as a result of electron excursion from MOF to BPNS component via strong Zn–P interfacial interaction. The strong Zn–P interaction may influence the intrinsic growth habit of MOF, leading to the oriented growth of the (112) plane and the formation of the energy-stable interface of BP(020)–MOF(112).

**Fig. 3:**
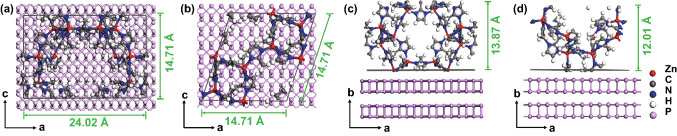
Atomic arrangement of the (011) and (112) planes of a single MOF unit cell. **a** and **b** show top views of the (112) and (011) plane. **c** and **d** show side views of the (112) and (011) plane

The priority of the MOF (112) plane orientation over the MOF (011) plane growing on the (020) plane of the BPNSs can be explained from two key points: zinc atom density and steric hindrance. Firstly, since the Zn–P interaction is the direct force determining the orientation growth of MOF on the BPNSs, the plane holding a higher Zn atom density with a stronger Zn–P interaction will be preferentially formed at the interface between MOF and the BPNSs. Figure 3a, b shows the top views of MOF (112) plane and MOF (011) plane cut out from single MOF unite cell. Calculations suggest that (112) plane has a higher Zn atom density than (011) plane (Table S1). We also analyze the Zn atom density of the (002), (013) and (222) planes which are most seen in the XRD pattern (Fig. S6). The result reveals that (112) plane has the highest Zn atom density among the above planes (Table S1). A higher Zn atom density with more Zn atoms in a given area of the MOF layer enables stronger Zn–P interaction to form a stabler BP-MOF interface. Secondly, the (112) plane has no steric hindrance for Zn–P interaction, yet the (011) plane suffers from a large steric hindrance. As shown in Fig. 3c, d, Zn atoms of the (112) plane are directly exposed and can approach P atoms to allow strong Zn–P interaction, while Zn atoms of the (011) plane are hindered by imidazole rings, which makes it difficult to directly interact with P atoms of the BPNSs. Take all into account, the MI layer with (112) plane dominant was induced by the strong Zn–P interaction, which results in the stable interface of BP(020)-MOF(112) that are crucial for high-performance electrochemical potassium storage.

### Electrochemical Measurements

**Fig. 4:**
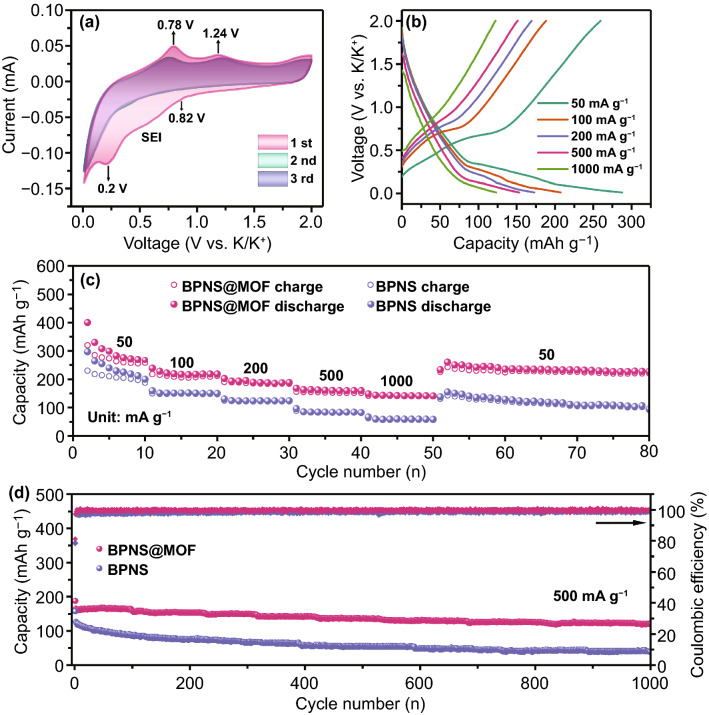
**a** CV profiles of the BPNS@MOF electrode at a scan rate of 0.1 mV s^−1^ for KIB. **b** Charge–discharge curves of the BPNS@MOF electrode in KIB. **c** Rate performance of the BPNS@MOF and BPNS electrodes in KIB at different current densities from 50 to 1000 mA g^−1^. **d** Cycle performance of the BPNS@MOF and BPNS electrodes in KIB at the current density of 500 mA g^−1^

Electrochemical behaviors of the BPNS@MOF and BPNS as KIB anodes were investigated first. The cyclic voltammetry (CV) curves of the BPNS@MOF are shown in Fig. 4a. During the initial cathodic scan, a broad peak located at 0.4–0.75 V is ascribed to the decomposition of electrolyte and the formation of solid electrolyte interface (SEI) film. Two peaks located at 0.82 and 0.2 V correspond to the stepwise potassiation of BP to form the final K_4_P_3_ phase. In the anodic sweep, two peaks located at 0.78 and 1.24 V reveal the stepwise depotassiation process [22, 23]. Compared to the BPNS electrode (Fig. S7), CV curves of the BPNS@MOF electrode are well overlapped in the subsequent cycles and show a higher reversibility. The HRTEM image (Fig. S8) coupling with cyclic voltammetry (CV) curves (Figs. 4a and S7) prove that both BPNS@MOF and BPNS anodes undergo the typical alloying reactions with *K* ions and the final alloying product is K_4_P_3_. The charge–discharge curves of the BPNS@MOF electrode in Fig. 4b exhibits obvious plateaus and slope shape compared to that of the BPNS electrode (Fig. S9), indicating the sufficient depotassiation/potassiation reaction of BP in the BPNS@MOF electrode. Specifically, a sloping region from 1.0–0.4 V followed by an inclined plateau from 0.4–0.1 V is observed in the discharge profile of the BPNS@MOF electrode, which corresponds to the stepwise potassiation process of BP to form the final K_4_P_3_ phase. In the charge process of the BPNS@MOF electrode, an inclined plateau from 0.4–0.75 V followed by a sloping region up to 2.0 V corresponds to the stepwise depotassiation reactions of BP. Moreover, the BPNS@MOF electrode manifests better rate capability compared with the BPNS electrode as shown in Fig. 4b, c and Table S2. The capacities achieved for the BPNS@MOF electrode (Fig. 4b) at the same current density are obviously higher than that of the BPNS electrode (Fig. S9). Relatively high average capacities of 301, 222, 192, 162 and 143 mAh g^−1^ are delivered at respective current densities of 50, 100, 200, 500 and 1000 mA g^−1^ (Fig. 4c, Table S2). Moreover, when the current density finally returns back to 50 mA g^−1^, a capacity of 261 mAh g^−1^ is still achieved for the BPNS@MOF electrode, with a high capacity retention of 97.2% (compared with the 10^th^ cycle at 50 mA g^−1^). The values are much higher than that for the BPNS electrode (155 mAh g^−1^ at 50 mA g^−1^, with a capacity retention of 77.6%), manifesting an excellent rate capability of the BPNS@MOF electrode. Furthermore, the BPNS@MOF electrode also exhibits excellent cycling stability. The BPNS@MOF electrode can maintain a capacity of 121 mAh g^−1^ at a higher current density of 500 mA g^−1^ with a high capacity retention of 73% after 1000 cycles (Fig. 4d). In contrast, the BPNS electrode shows a lower capacity of 43 mAh g^−1^ as well as a low capacity retention of 34%. Moreover, the BPNS@MOF electrode still remains electrode integrity after cycles and exhibits a smaller thickness change ratio (33.8%) than the BPNS electrode (47.1%) (Fig. S10). The results demonstrate that the MI layer can effectively suppress electrode expansion for stable potassium storage. The performance enhancement is ascribed to the decreased charge transfer resistance at the electrode/electrolyte interface which may facilitate a better charge transfer capability and electrochemical kinetics (Fig. S11, Table S3).

**Fig. 5:**
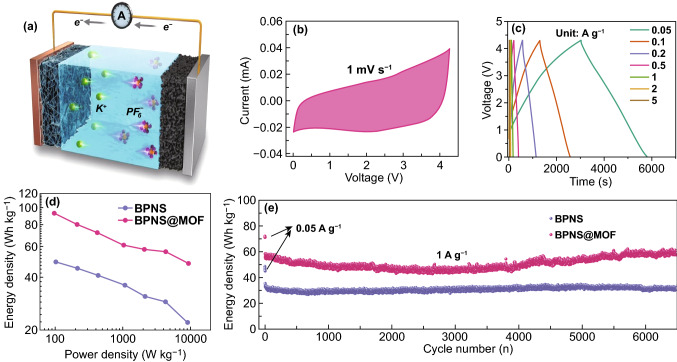
**a** Schematic configuration of a KIC device. **b** CV curve of the BPNS@MOF electrode at scan rate of 1 mV s^−1^. **c** Galvanostatic charge/discharge curves of the BPNS@MOF//AC full cells at different current densities. **d** Ragone plots of the BPNS//AC and BPNS@MOF//AC electrodes for KIC. **e** Cyclic performance of the BPNS//AC and BPNS@MOF//AC electrodes for KIC

Asymmetric hybrid K-ion capacitors using the BPNS@MOF or BPNS as the anode and commercial activated carbon (AC, Fig. S12) as the cathode were assembled, as illustrated in Fig. 5a. Based on the voltage range of the CV tests in half-cell configuration with metallic potassium as the counter electrodes (Figs. 4a and S13), the potential window of 0 − 4.3 V was selected for the KIC full cell (Fig. 5b). For the KIC assembly, the mass ratio between the AC cathode and BP anode was optimized to be 4: 1 on a basis of the charge balance theory (Q^+^ = Q^−^) (Fig. S14a). Figure 5c exhibits the galvanostatic charge–discharge profiles ranging from 50 to 5000 mA g^−1^. The voltage profiles at all current densities are somewhat different from the linear behavior for an ideal supercapacitor, mainly due to the combination of charge storage mechanisms based on faradaic and non-faradaic reactions. The energy/power densities of KICs are shown in a Ragone plot in Fig. 5d. The BPNS@MOF//AC full cell can deliver a high energy density of 93 Wh kg^−1^ at a power density of 97 W kg^−1^ and a moderate energy density of 48 Wh kg^−1^ at a high power output of 9380 W kg^−1^. In contrast, BPNS//AC capacitor delivers a maximum energy density of 49 Wh kg^−1^ at a power density of 102 W kg^−1^ and maintains 22 Wh kg^−1^ at 92 W kg^−1^, which put up a much lower energy density than BPNS@MOF//AC at a similar power density. The cycle performance of KICs is shown in Fig. 5e, where the BPNS@MOF//AC capacitor can maintain an energy retention of 99.5% after 6500 cycles at a current density of 1 A g^−1^, much higher than that of the BPNS//AC capacitor (86%). Furthermore, BPNS@MOF samples with different MOF contents were also assembled into KICs and compared their performance (Fig. S14b, c). It is found that their combined energy/power delivery and cycle performance are worse than the typical BPNS@MOF sample. In addition, as shown in Fig. S15, the BPNS@MOF and BPNS@MOF-3 electrodes can remain electrode integrity after cycles due to the relatively thicker MOF interphase layer on BPNSs, while the BPNS@MOF-2 electrode shows obvious cracks on electrode surface after cycles (inset of Fig. S15d1 *vs.* insets of Fig. S15b1, f1), which may be due to that the thin MOF interphase layer on BPNS cannot effectively suppress electrode expansion. Notably, the BPNS@MOF exhibits a moderate electrode expansion ratio (13.6%), smaller than that of the BPNS@MOF-2 electrode (18.6%), yet larger than the BPNS@MOF-2 electrode (10.7%) (Fig. S15b *vs.* Fig. S15d, f). The result implies that the typical BPNS@MOF holds an optimal thickness of MOF coating layer to obtain the balance of potassium storage capacity and electrode structural stability. The EIS results for KIC show that the typical BPNS@MOF sample renders the lowest charge transfer resistance in KIC, which may facilitate a better charge transfer capability and electrochemical kinetics (Fig. S16). Moreover, performance comparison of the BPNS@MOF-based KIC and the state-of-the-art KICs is summarized in Fig. S17. The cycle performance outperforms most of the reported KICs so far (Fig. S17, Table S4). The results suggest that the BPNS@MOF with an ordered porous MI layer stands for an ideal anode material for potassium storage devices.

Moreover, we found that an ultrathin MI layer favors forming a thinner solid electrolyte interphase (SEI) film (3–3.2 nm) on the surface of the BPNS@MOF relative to that of the BPNSs (6.4–6.8 nm), as shown in Fig. S18. The thinner SEI can facilitate ion insertion/extraction for stable energy storage, as demonstrated in our previous work [45]. Besides, it is analyzed that K_2_SO_4_ is the main component and accounts for the largest percentage among the inorganic components for the BPNS electrode cycled in both KIB and KIC (Figs. S19–S22). The chemical and structural stability of the MI layer in the BPNS@MOF during electrochemical potassium storage was also investigated in depth (Figs. S23 and S24). The result evidences that MI layers can be well preserved upon cycling in KIBs and KICs.

### Calculation and Analysis of Reaction Kinetics

**Fig. 6:**
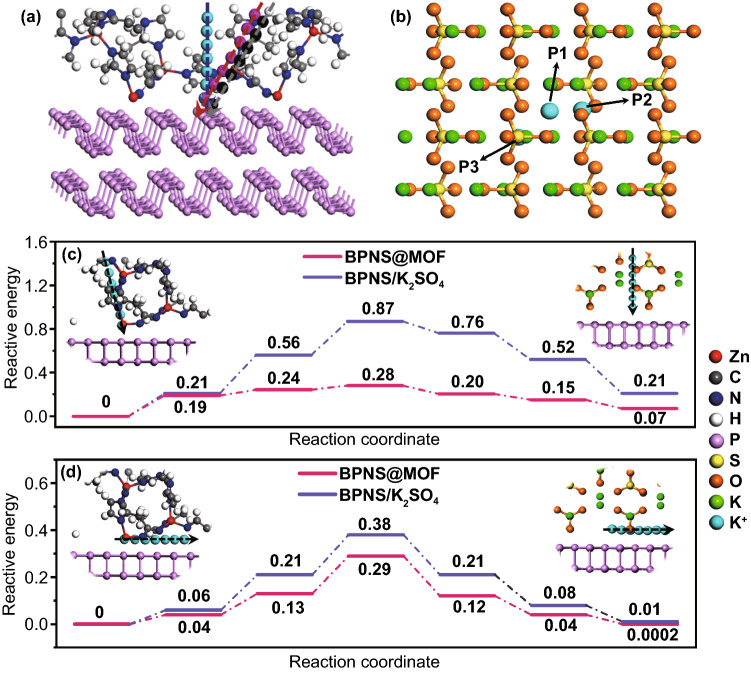
Theoretical analysis of reaction kinetics. **a** Different pathways when K ions pass through MOF in the model of BP(020)-MOF(112). **b** Different sites of K ions in the model of BP(020)-K_2_SO_4_ (002). (BPNS is neglected in order to exhibit the site clearly). **c** Diffusion energy profile for potassium diffusion through the MOF and K_2_SO_4_. Inset of **c** is the side views of the diffusion pathways. **d** Diffusion energy profile for potassium over the surface of BP. Inset of **d** is the side views of the diffusion pathways. Inset of both **c** and **d** shows *ab* plane

To quantitatively analyze the *K* ion diffusion behavior induced by the MI layer, GITT was employed to determine the reaction kinetics of the BPNS@MOF anodes (Fig. S25). The GITT result demonstrates that the BPNS@MOF electrode can achieve a faster K^+^ migration compared to the BPNS electrode. The increment of the diffusion coefficient may be attributed to the ordered porous structure of the MI layer which provides direct and efficient diffusion pathways of potassium ions during cycles. It is well known that SEI is a passivation layer formed on electrode surfaces from decomposition products of electrolytes [46]. Ideally, the SEI layer should facilitate reversible ions transport and prevent electrode pulverization by suppressing excessive interfacial interactions. However, the SEI layer is normally formed in a dense or nonporous/irregularly porous structure, which is adverse to ion diffusing across to react with electrode materials. For the two systems of the BPNS electrode with the thicker SEI layers and the BPNS@MOF electrode with the thinner SEI and ultrathin MI layers (Fig. S18), the MI layer in the BPNS@MOF in position is analogous to the part of the thicker SEI layer, but may play distinct role in reaction kinetics. To further bring insight into the improved depotassiation/potassiation kinetics by the ordered porous MI in the BPNS@MOF over the corresponding part of SEI on the BPNS electrodes, we carried out calculation of K diffusion energy barriers for the two systems using density functional theory (DFT) [47]. It is noteworthy that for the BPNS system, K_2_SO_4_ as the main inorganic component of SEI (Figs. S19–S22) was selected to represent the naturally generated SEI to make a comparison with MI layer. The models used for theoretical calculations are illustrated in Fig. S26a, b, where BP(020)–MOF(112) interface is employed for the BPNS@MOF, while BP(020)–K_2_SO_4_ (002) is employed for the BPNSs. Calculations of *K* diffusion energy barriers for the models of both BPNS@MOF and BPNS/K_2_SO_4_ are divided into two parts, that is, the migration pathway of (i) *K* ions across MOF or K_2_SO_4_ layer and (ii) *K* ions along the surface of BP nanosheet. The optimal migration pathways for the two systems are illustrated in Fig. S26c, d, which are determined by identifying the lowest energy barrier of several possible pathways in MOF layer (Figs. 6a and S27, S28), or the energetically favorable adsorption site in K_2_SO_4_ layer (Figs. 6b and S29). It is noted that for the pathway (ii), *K* along the zigzag direction of BPNSs surface is adopted for the both systems of BPNS@MOF and BPNS/K_2_SO_4_, which turns out to be the optimum pathway with the lowest diffusion energy barriers [48, 49]. Diffusion energy profiles for *K* ions diffusion pathway (i) through the MOF or K_2_SO_4_ and (ii) along the surface of BP are shown in Fig. 6c and 6d, respectively. The energy barriers of *K* ion diffusion through BPNS@MOF for pathways (i) and (ii) are both lower than those through BPNS/K_2_SO_4_. The result indicates that the ordered porous MI layer is more favorable than K_2_SO_4_ for *K* ions to pass through, thus accounting for a better rate performance.

## Conclusions

In summary, ultrathin MI layers with ordered pores and high stability have been constructed on the surface of the BPNSs, which acting as a protective layer, is proved to play a significant role in improving the electrochemical potassium storage performance. When employed as an anode for KIC, the BPNS@MOF electrode exhibits remarkable performance compared with the pure BPNS electrode. The performance enhancement is ascribed to facilitated K-ion diffusion and reinforced electrode stability enabled by the MI layer with the porous and robust interface. This study provides a new strategy to regulate reaction kinetics and stability of electrode materials through MOF-based interface engineering.

## Supplementary Information

Below is the link to the electronic supplementary material.

Supplementary file 3 (PDF 2398 kb)
