# Supplementary material for: Armoring Black Phosphorus Anode with Stable Metal–Organic-Framework Layer for Hybrid K-Ion Capacitors
Source: Nanomicro Lett. 2021 Jan 4;13:42. doi: 10.1007/s40820-020-00570-7 (PMC8187701; doi:10.1007/s40820-020-00570-7)
Supplement: Supplementary file 1 — Supplementary file 3 (PDF 2398 kb) [file 40820_2020_570_MOESM1_ESM.pdf]

Supporting Information for

Armoring Black Phosphorus Anode with Stable Metal-Organic-Framework Layer for Hybrid K-Ion Capacitors

Mengzhu Xu[1, 2, #], Yutong Feng[1, 2, #], Bingjie Chen[2], Ruijin Meng[2], Mengting Xia[2], Feng Gu[3], Donglei Yang[4], Chi Zhang[1, 2, *], Jinhu Yang[1, 2, *]

[1]Research Center for Translational Medicine & Key Laboratory of Arrhythmias of the Ministry of Education of China, East Hospital, Tongji University School of Medicine, Shanghai 200120, P. R. China

[2]School of Chemical Science and Engineering, Tongji University, Shanghai 200092, P. R. China

[3]Institute for Process Modelling and Optimization, Jiangsu Industrial Technology Research Institute, 388 Ruoshui Road, SIP, Suzhou, Jiangsu, P. R. China

[4]Institute of Molecular Medicine School of Medicine, Shanghai Jiao Tong University, Shanghai 200127, P. R. China

[#]M. Z. Xu and Y. T. Feng contributed equally to this work

*Corresponding authors. E-mail: chizhang@tongji.edu.cn (Chi Zhang); yangjinhu@tongji.edu.cn (Jinhu Yang)

Supplementary Figures and Tables

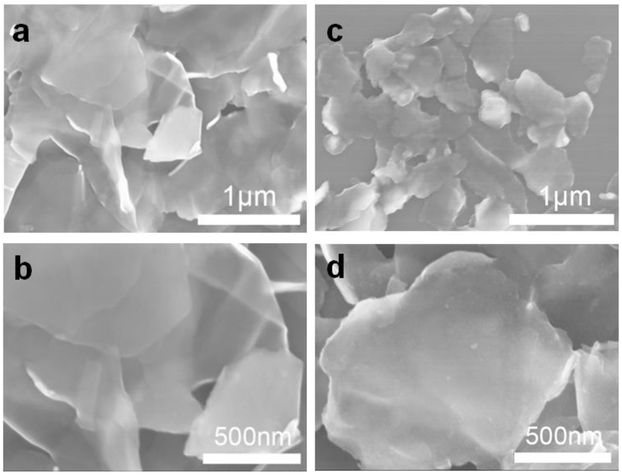

Fig. S1 SEM images of (**a, b**) the BPNSs and (**c, d**) the BPNS@MOF

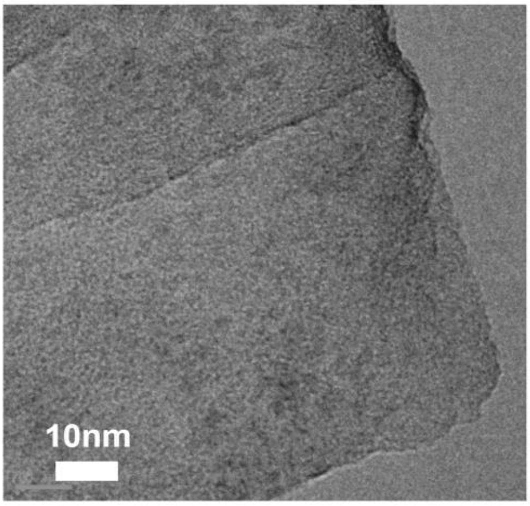

Fig. S2 High magnification TEM image of the BPNS@MOF

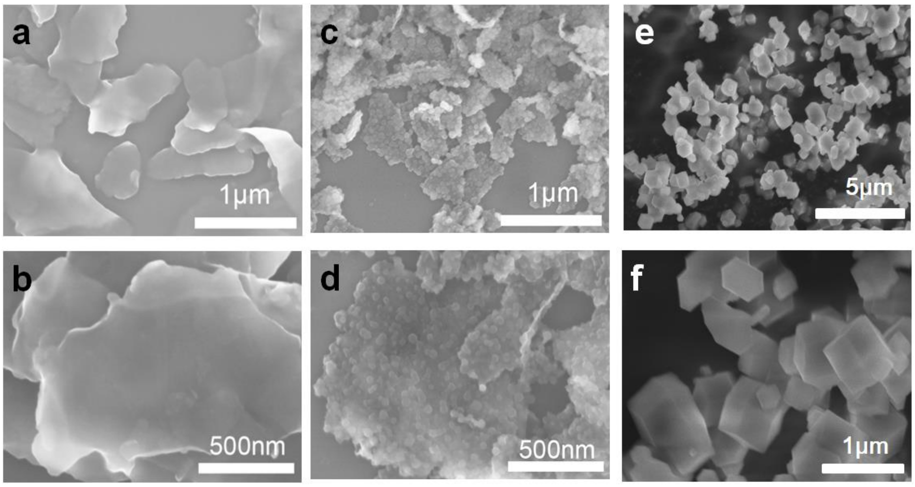

Fig. S3 SEM images of (**a, b**) the BPNS@MOF-2, (**c, d**) BPNS@MOF-3 and (**e, f**) ZIF-8

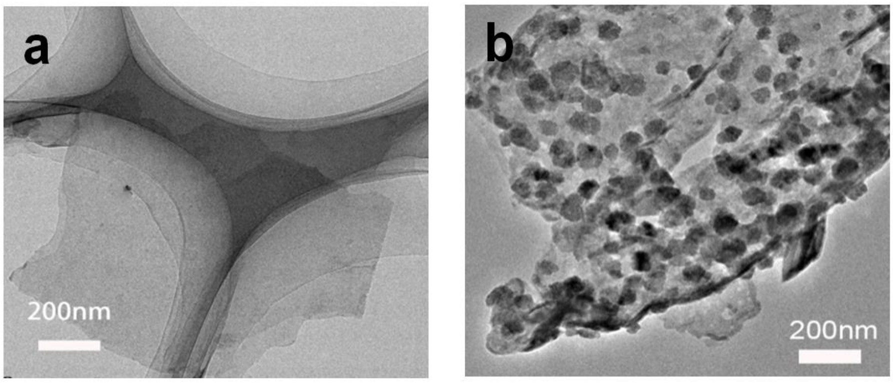

Fig. S4 TEM images of (**a**) the BPNS@MOF-2 and (**b**) BPNS@MOF-3

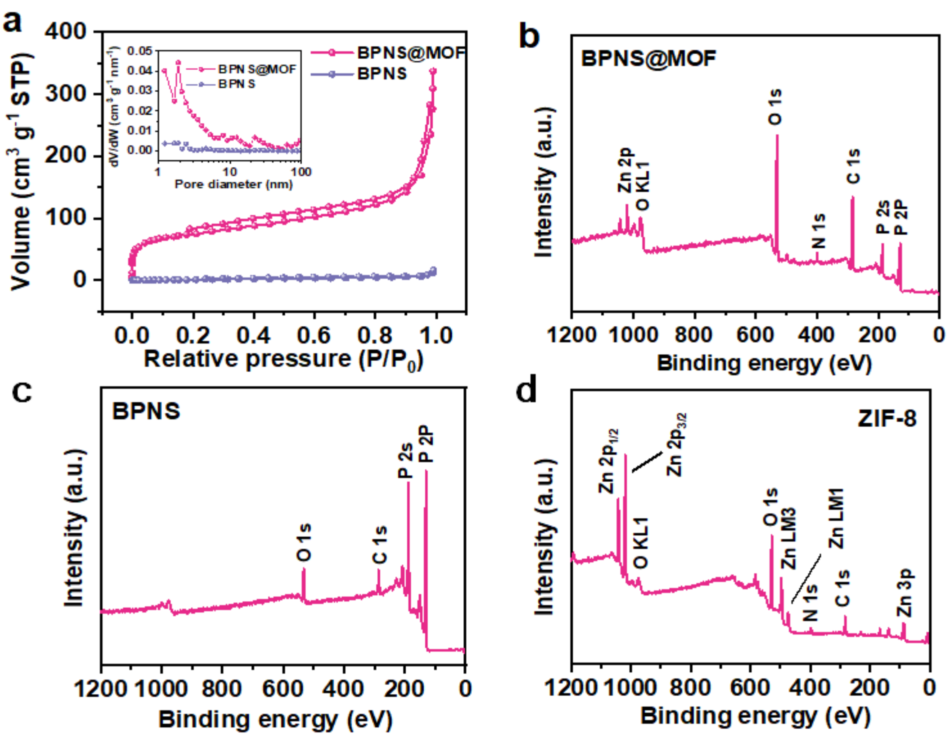

Fig. S5 (**a**) N_2 adsorption-desorption isotherms of the BPNS@MOF and the BPNSs. (**b-d**) XPS survey spectra of the BPNS@MOF, BPNSs, and ZIF-8

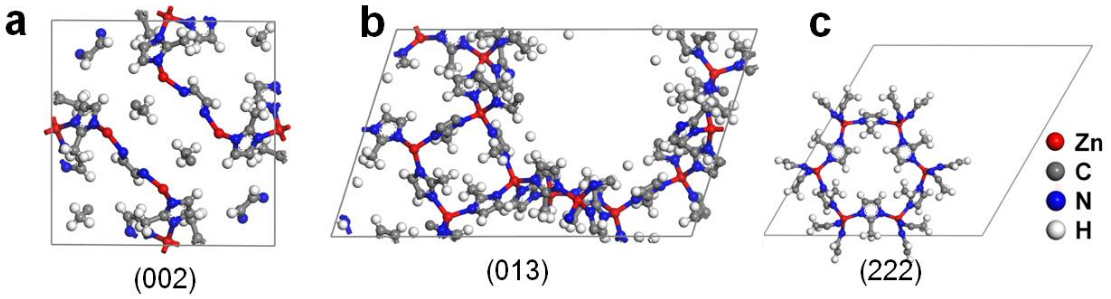

Fig. S6 Top views of different planes of (002), (013) and (222) of ZIF-8

Note: Comparison of Zn atom density of different planes is listed in Table S1, and the result shows that (112) plane has the highest Zn atom density among different planes of (011), (002), (112), (013) and (222), which are most commonly seen in the XRD pattern. The results demonstrate that the higher interaction between Zn and P caused by relatively high Zn density drives the orientation of the dominant plane from (011) to (112).

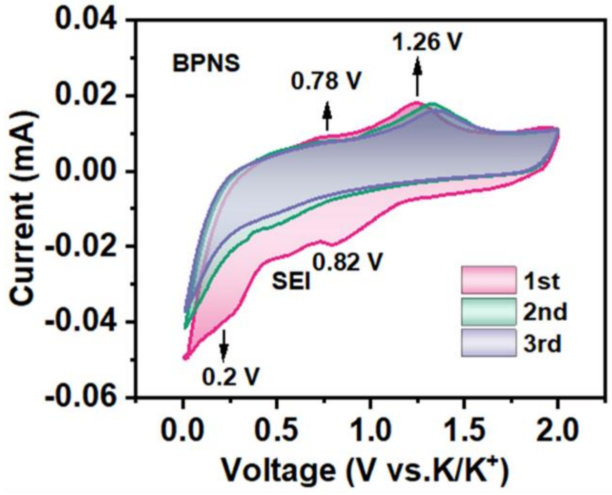

Fig. S7 CV profiles of the BPNS electrode at a scan rate of 0.1 mV s^{-1} for KIB

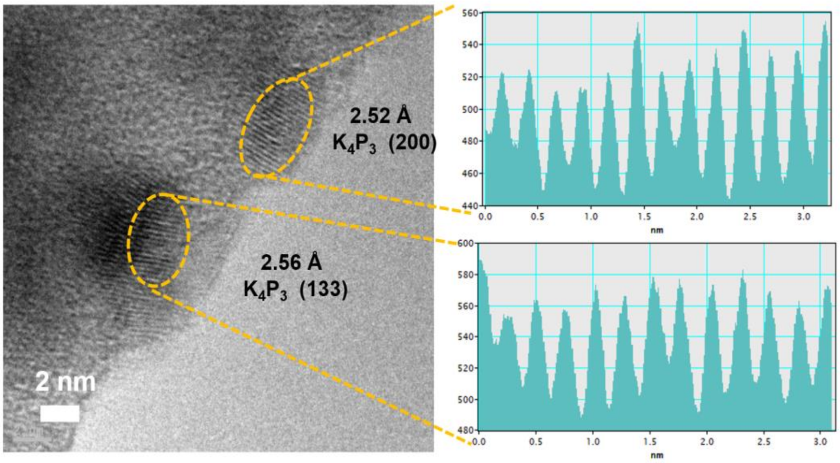

Fig. S8 HRTEM image with crystal lattice analysis of the BPNSs after the 10 cycles in KIB at a terminal voltage of 0.01 V

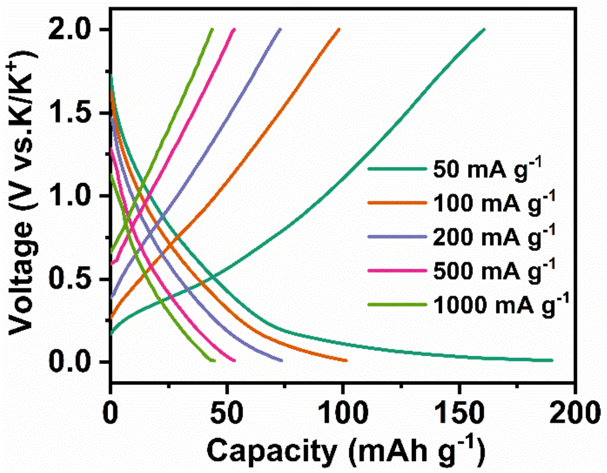

Fig. S9 Charge–discharge curves of the BPNS electrode in KIB

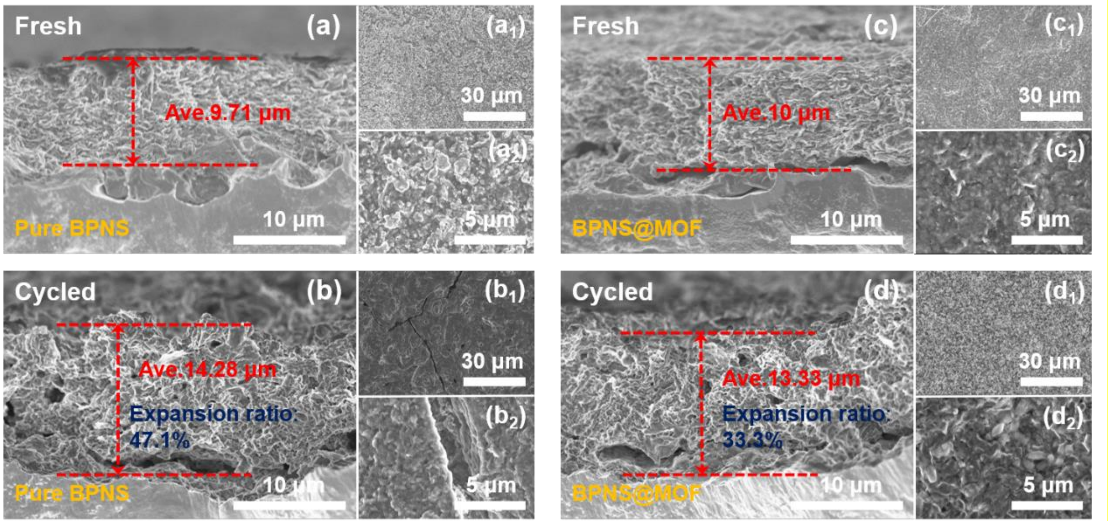

Fig. S10 (**a-d**) Comparison of electrode thickness and expansion ratio between the BPNS electrode (**a,b**) and the BPNS@MOF electrode (**c,d**) before cycling and after cycles in KIB. Insets in Figure S10 (**a_1-d_2**) are the top view of electrode with different magnifications.

Note: The BPNS@MOF electrode still remains electrode integrity after cycles while the BPNS electrode shows obvious cracks on electrode surface (inset of Fig. S10d *vs* inset of Fig. S10b). Moreover, the BPNS@MOF electrode exhibits a smaller thickness change ratio (33.8%) than the BPNS electrode (47.1%) (Fig. S10d *vs* Fig. S10b).

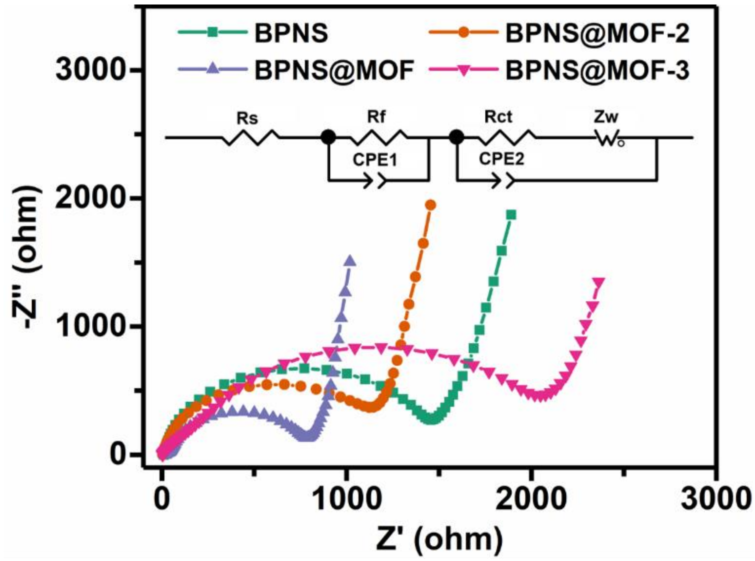

Fig. S11 Electrochemical impedance spectra of the BPNS electrode and the BPNS@MOF electrodes with different MOF contents for KIBs before cycling. Inset shows the corresponding equivalent circuit.

Note: As shown in Fig. S11, the BPNS electrode and the BPNS@MOF electrodes with different MOF contents consists of R_s (represents the internal resistance of the test battery), R_f and CPE_1 (assigned to the resistance and constant phase element of

SEI film), R_{ct} and CPE_2 (associate with the charge-transfer resistance and constant phase element of the electrode/electrolyte interface), and Z_W (ascribe to Warburg impedance corresponding to the K^+ diffusion process). From the fitted values shown in Table S3, it can be clearly seen that the values of R_f of the BPNS@MOF electrodes (with different MOF contents) are much lower than that of the BPNS electrode, indicating the MOF interphase layer can reduce the resistance at the interface between the SEI film and the electrolyte. Moreover, the BPNS@MOF electrode offers the lowest R_{ct} value of 750 Ω compared to the others that accounts for the better charge transfer capability and electrochemical kinetics.

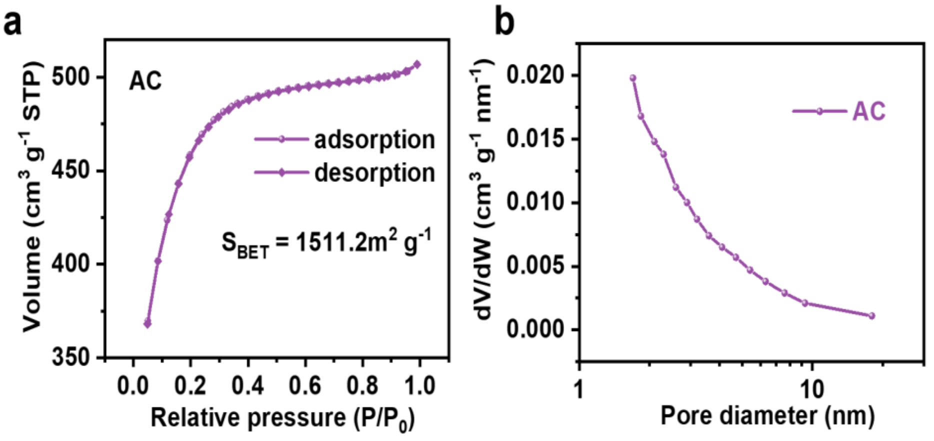

Fig. S12 (**a**) N_2 adsorption-desorption isotherms and (**b**) pore size distribution of activated carbon (AC)

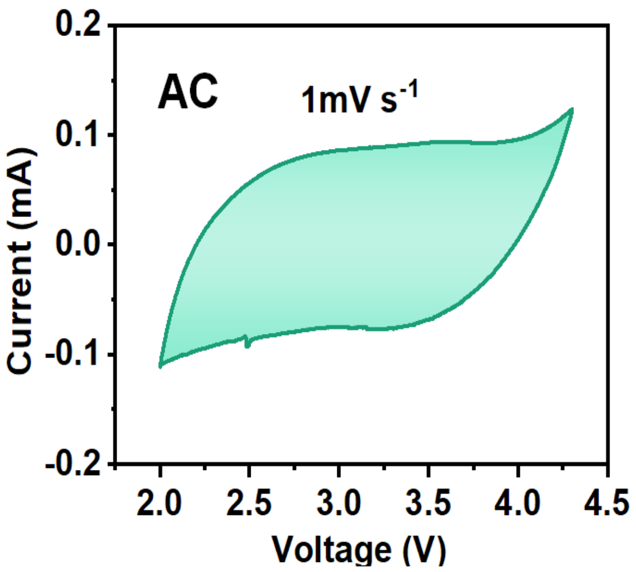

Fig. S13 CV test of AC in half-cell configuration with metallic potassium as the counter electrodes

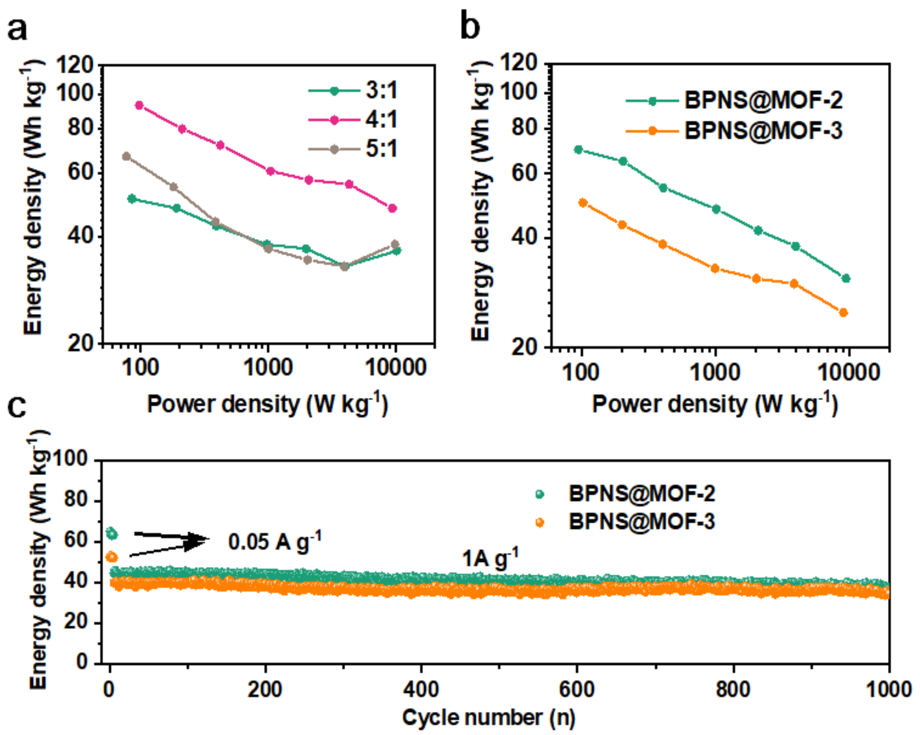

Fig. S14 KIC performance. (**a**) Ragone plots of the BPNS@MOF//AC capacitors with different cathode to anode mass ratios. (**b, c**) Ragone plots and cycling performance of BPNS@MOF-2//AC and BPNS@MOF-3//AC capacitors

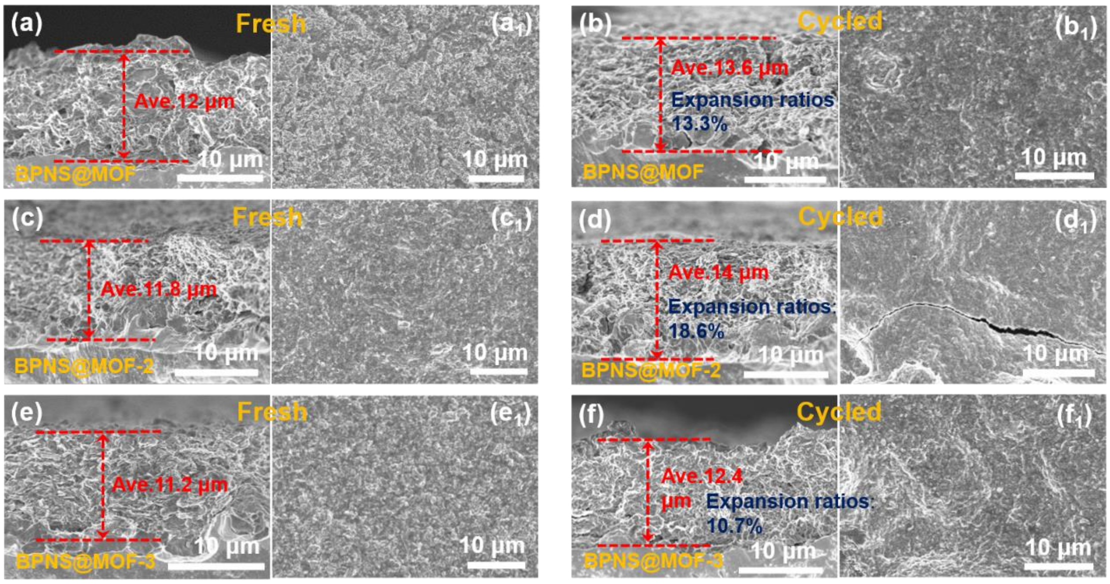

Fig. S15 a-d Comparison of electrode thickness and expansion ratio among the BPNS@MOF electrode (**a, b**), the BPNS@MOF-2 electrode (**c, d**) and the BPNS@MOF-3 electrode (**e, f**) before cycling and after 100 cycles in KIC. Insets in Fig. R2 (**a_1-f_1**) are the top views of the three electrodes with different magnifications.

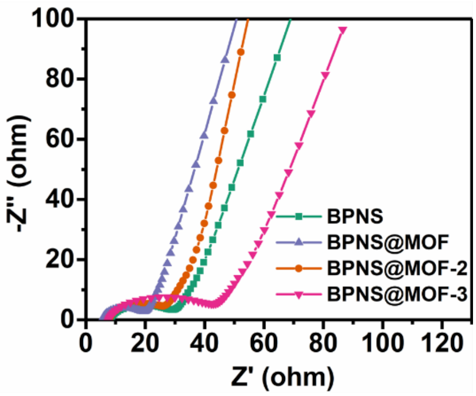

Fig. S16 Electrochemical impedance spectra of the BPNS electrode and the BPNS@MOF electrodes with different MOF contents for KICs before cycling

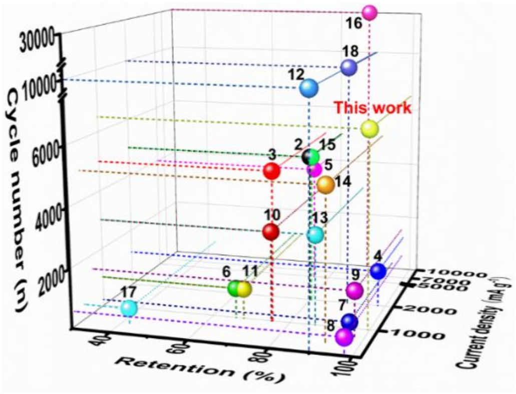

Fig. S17 Comparison of cycle performance of the BPNS@MOF//AC KICs and the state-of-the-art KICs. (The performance of all these works calculated based on the total mass of both anodes and cathodes)

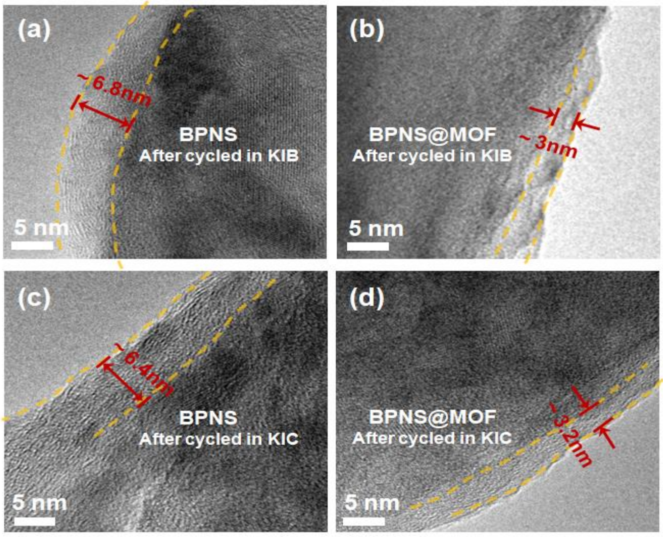

Fig. S18 (**a, b**) HRTEM images of the BPNSs (**a**) and the BPNS@MOF (**b**) after 10 cycles in KIB, respectively. (**c, d**) HRTEM images of the BPNSs (**c**) and the BPNS@MOF (**d**) after 10 cycles in KIC

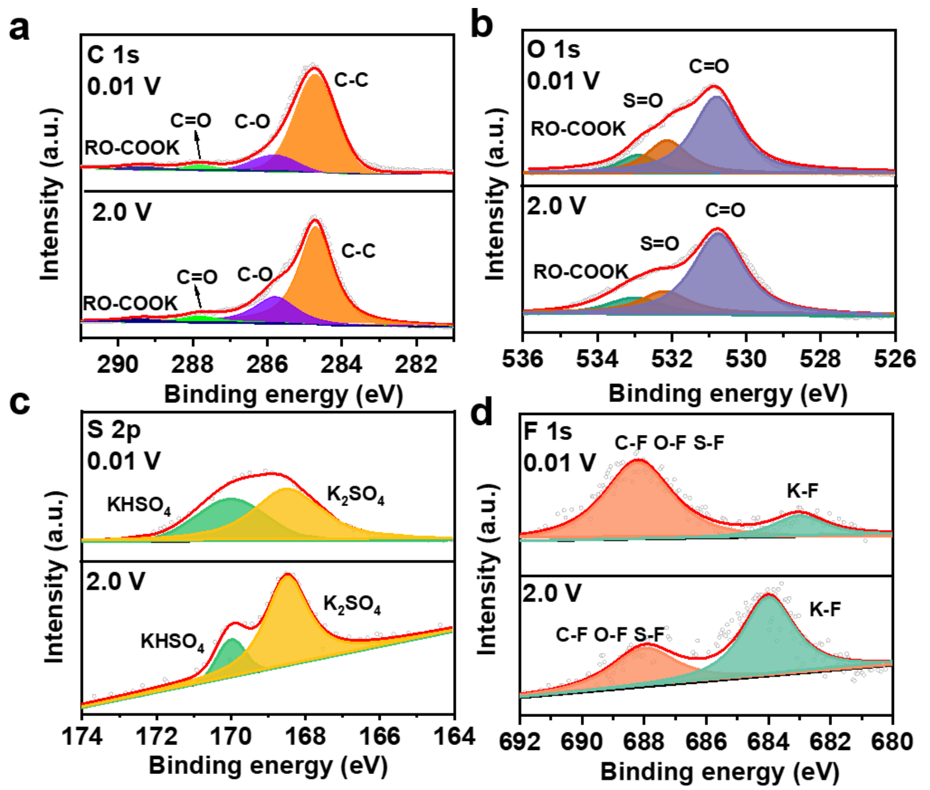

Fig. S19 XPS spectra of (**a**) C 1s, (**b**) O 1s, (**c**) S 2p, (**d**) F 1s of the BPNS electrode in KIB after the 1st cycle

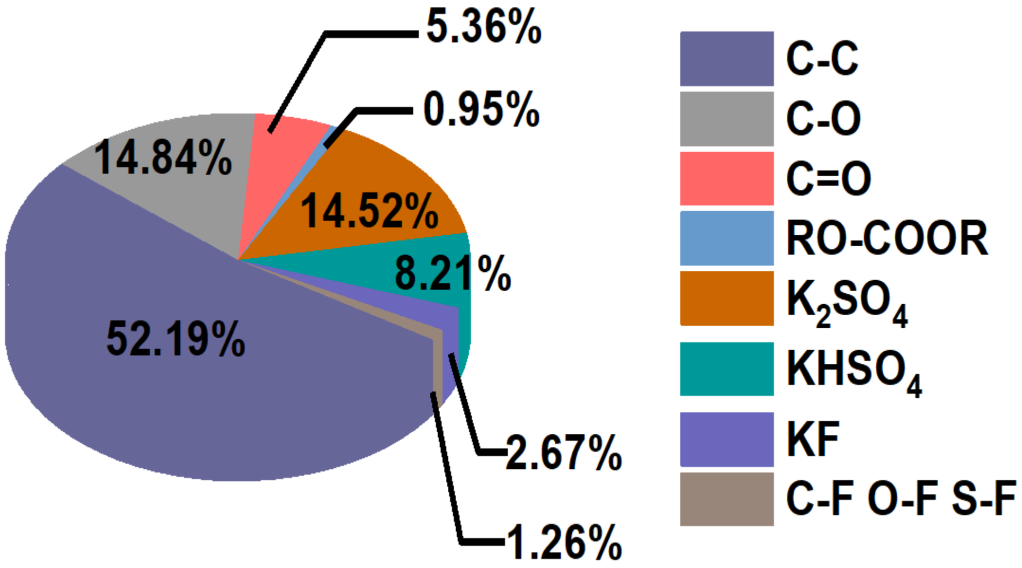

Fig. S20 Relative content of different species in SEI layers. The conclusion based on the data of Fig. S19.

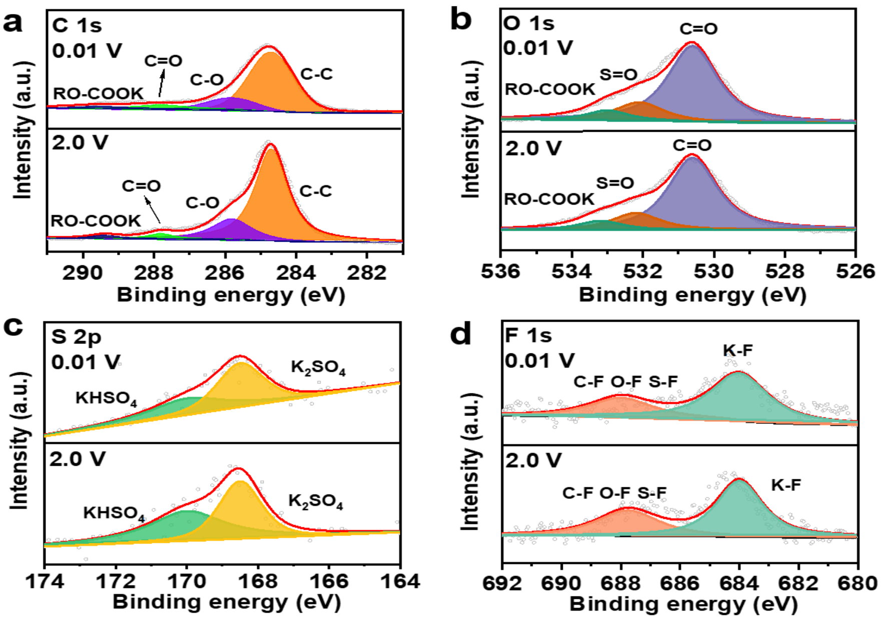

Fig. S21 XPS spectra of (**a**) C 1s, (**b**) O 1s, (**c**) S 2p, (**d**) F 1s of the BPNS electrode in KIB after 10 cycles

Note: The position and relative intensity of the peaks in XPS spectra of C 1s, O 1s, S 2p, and F 1s keep basically unchanged compared with that in the 1st cycle, indicating the composition of SEI is the same.

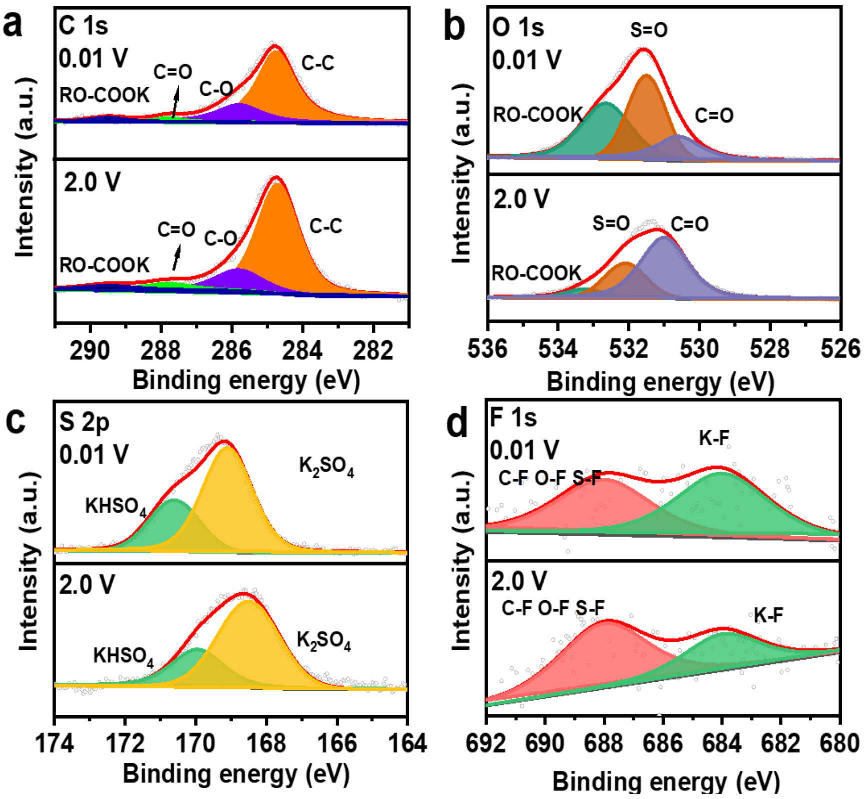

Fig. S22 XPS spectra of (**a**) C 1s, (**b**) O 1s, (**c**) S 2p, (**d**) F 1s of the BPNS electrode in KIC after 10 cycle

SEI component analysis (Fig. S19-S22)

XPS measurements were performed to analyze the composition of SEI layers on the surface of BPNS anodes.

First of all, we analyzed XPS spectrum of C, O, S and F in the 1st cycle (Fig. S19) with cut off voltage of 0.01V. The C 1s spectrum can be split into four peaks with binding energies of 284.8 (C-C), 285.8 (C-O), 287.8 (C=O) and 289.5 eV (RO-COOK) [S19, S20]. C-C bonded carbon is ascribed to the conductive additive, and the C−O, C=O bonded carbon and RO-COOK species in the surface layer should originate from the decomposition and reduction of EC and DEC solvent. In O 1s spectra, the high intensity peak at 530.8 eV is attributed to C=O bonds while the peak at 532.3 eV is ascribed to S=O and the peak at 533eV should originate from RO-COOR [S21]. The S 2p spectra exhibit two peaks around 168.5 (K_2SO_4) and 170 ($KHSO_4$), further demonstrate salt reduction in the KFSI-based electrolyte [S22]. It was discovered that the F 1s spectrum shows two different fluorine species, which can be assigned to C-F, O-F and S-F species at 687.7 eV, due to incomplete decomposition of the salts, and KF species at a lower binding energy of 682.9 eV, which may be from the reduction of S-F bonds in the FSI^- [S23, S24].

In order to evaluate the composition of SEI layers, we calculated the integral areas of individual peaks and the normalized content of each component is summarized in Fig. S20. It can be obviously seen that K_2SO_4 accounts for the largest percentage among the inorganic components of SEI.

When the cell charge to 2.0 V (Fig. S19), no new components are observed. Even after the BPNS electrode was cycled for 10 cycles (Fig. S21), the XPS spectra show almost negligible changes at the cut off voltage of both 0.01 V and 2.0 V compared with the 1st cycle in Fig. S19. This means that a SEI film with a stable composition is formed on the surface of the BPNS electrode. What's more, the XPS spectra of the BPNS electrode cycled in KIC (Fig. S22) is similar with that cycled in KIB, which means there is no difference in the component of SEI formed in KIB and KIC.

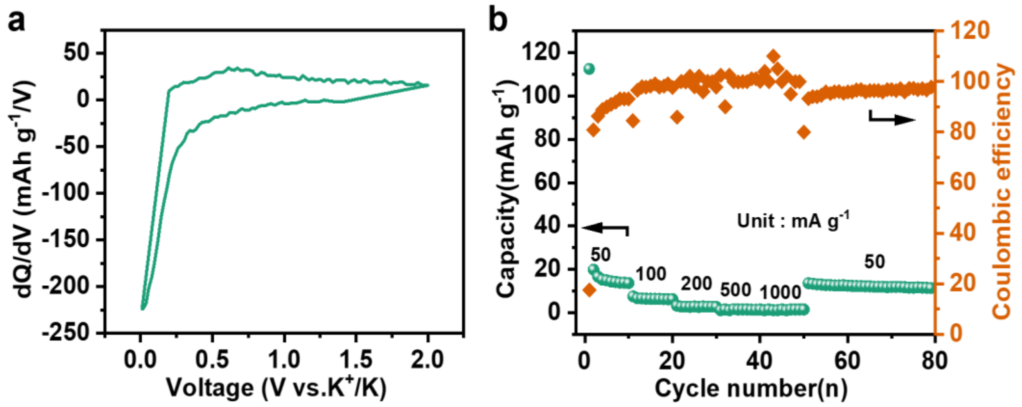

Fig. S23 (**a**) dQ/dV plot of the 3rd cycle during discharge process and charge process for ZIF-8 at 50 mAh g^{-1}. (**b**) Rate performance of ZIF-8 at different current densities

Note: As shown in Fig. S23a, the dQ/dV plot of the BPNS@MOF electrode doesn't display visible peak, indicating that ZIF-8 is almost electrochemical inert to potassium. Moreover, as shown in Fig. S23b, the BPNS@MOF electrode delivers a capacity as low as 14 mAh g^{-1} at a low current density of 50 mAh g^{-1}, and the capacity is decreased nearly to 0 mAh g^{-1} when the current is increased to 200 mAh g^{-1}. The rate performance of the ZIF-8 electrode is in good agreement with the result from the dQ/dV plot, confirming ZIF-8 is almost electrochemical inactive in KIBs.

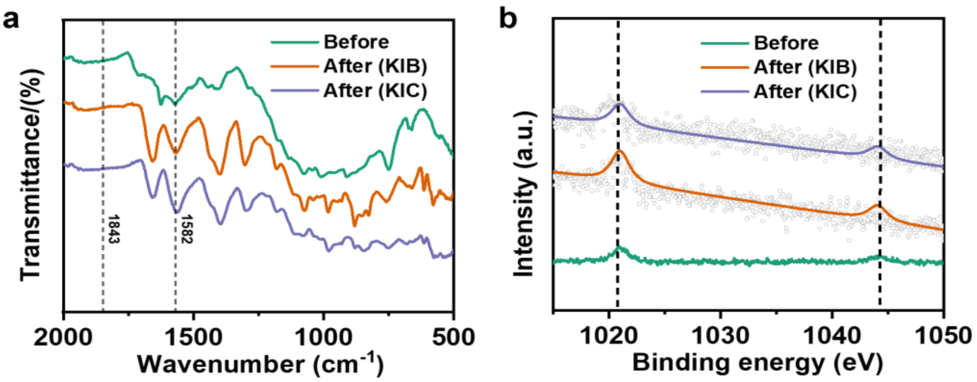

Fig. S24 (**a**) FTIR spectra and (**b**) XPS spectra (Zn 2p) of the BPNS@MOF electrode before cycling and after 100 cycles in KIB and KIC

On the other hand, FTIR and XPS measurements testify that the MI layers in the BPNS@MOF also exhibit high structural stability and can preserve in the process of electrochemical potassium storage. Figure S24a shows the FTIR spectra of the BPNS@MOF electrodes in KIB and KIC before cycling and after 100 cycles. The FTIR peak at 1580 cm^{-1} belonging to C=N stretching vibration [S25] still exists after cycled in both KIB and KIC, suggesting the structural preservation of the imidazole ring skeleton. Besides, there is the no peak appearing at 1843 cm^{-1} which is assigned to the resonance between the N–H···N bending "out of plane" and N–H stretching vibrations [S26, S27], indicating that protonation of N in imidazole ring is not occurred and the 2-methylimidazole still coordinates with Zn ions. In addition, the corresponding Zn 2p XPS spectra display that the binding energy of Zn $2p_{3/2}$, Zn $2p_{1/2}$ of the BP@MOF electrode keeps unchanged before and after cycling in both KIB and KIC (Fig. S24b), proving that Zn-N bonds in MOF is not broken during the charge-discharge process, similar to the case in lithium-ion batteries [S28]. All considered, MOF framework of ZIF-8, that is, MI layers can preserve in KIBs and KICs.

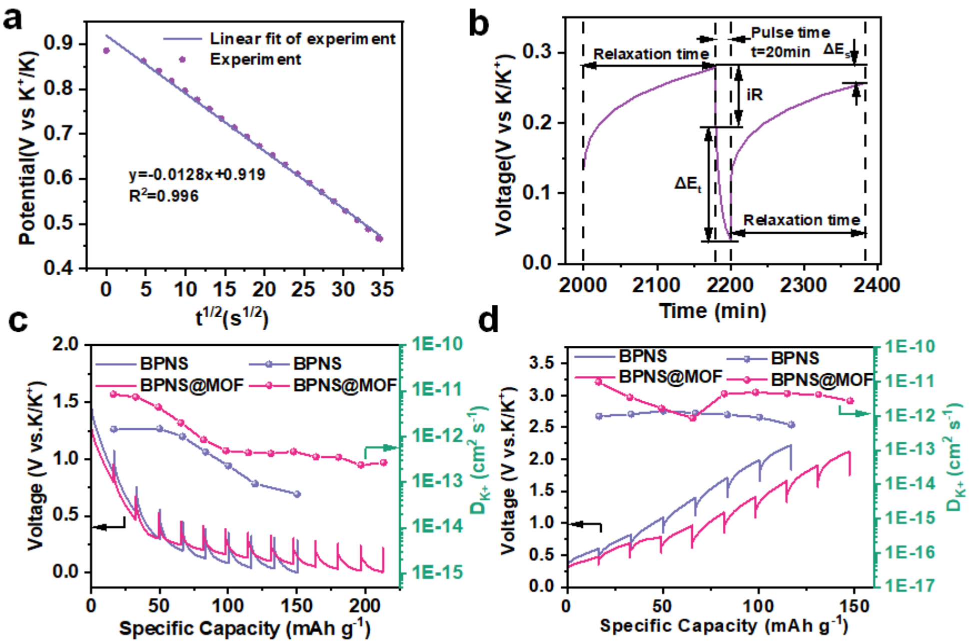

Fig. S25 (**a**) Liner behavior of E vs $\tau^{1/2}$ during current pulse in discharge process. (**b**) Transient voltage responses versus time profile of the BPNS@MOF electrode for a single GITT. (**c, d**) GITT profiles and the corresponding K^+ diffusion coefficient at the discharge process (**c**) and the charge process (**d**). Both curves were tested of the sixth cycle in the potential window of 0.01−2.0 V vs K^+/K (current density: 50 mA g^{-1}; time interval: 20 min; relaxation period: 3 h).

Note: The Galvanostatic intermittent titration technique (GITT) was first developed by Weppner and Huggins to determine the chemical diffusion coefficient of lithium in the Li_3Sb electrode and had been widely applied to systems of lithium ion batteries [S29], sodium ion batteries [S30], and potassium ion batteries [S31, S32]. Here, GITT was employed to determine the reaction kinetics of the BPNS@MOF anodes. We evaluated the diffusion coefficient of K^+ (D_k) by Fick's second law equation as the following equation:

$$D = \frac{4}{\pi\tau}\left(\frac{m_B V_M}{M_B S}\right)^2 \left(\Delta E_S / \tau\left(\frac{dE_\tau}{d\sqrt{\tau}}\right)\right)^2 \left(\tau \ll L^2/D\right) \qquad \text{(S1)}$$

Where τ is the duration of current pulse, m_B represents the mass of active material on the electrode, M_B and V_M are the molar mass and molar volume of active material, respectively, S is the surface area of the electrode, M_B/V_M is the gravimetric density of active material, L is the thickness of active material on the electrode. If the voltage has linear relation with $\tau^{1/2}$ (Fig. S25a), the Fick's second law equation above can be simplified to following equation:

$$D = \frac{4}{\pi\tau}\left(\frac{m_B V_M}{M_B S}\right)^2 \left(\Delta E_S / \Delta E_\tau\right) \qquad \text{(S2)}$$

Wherein, ΔEs is the difference in equilibrium potential before and after the current pulse, and ΔEτ is the variation of cell voltage caused by the impulse current. Figure S25b shows Transient voltage responses versus time profiles of the BPNS and BPNS@MOF electrodes after activation for 5 cycles, which is the original data to calculate diffusion coefficient D. All GITT curves were obtained in a potential window of 0.01-2V vs K^+/K. More detailed test information can be found in E vs t profile for a single GITT (Fig. S25c). Specifically, current of 50 mA g^{-1} was applied for 20 min followed by 3 h relaxation. In addition, $\bar{D}_K$ values of BPNS@MOF-2 and BPNS@MOF-3 are 9.86×10^{-13} and 3.09×10^{-13} cm^2 s^{-1} (Fig. S25d), respectively, which are lower than that of the typical BPNS@MOF. The difference confirms that the BPNS@MOF can achieve best reaction kinetics. This conclusion can also correspond to the previous electrical performance.

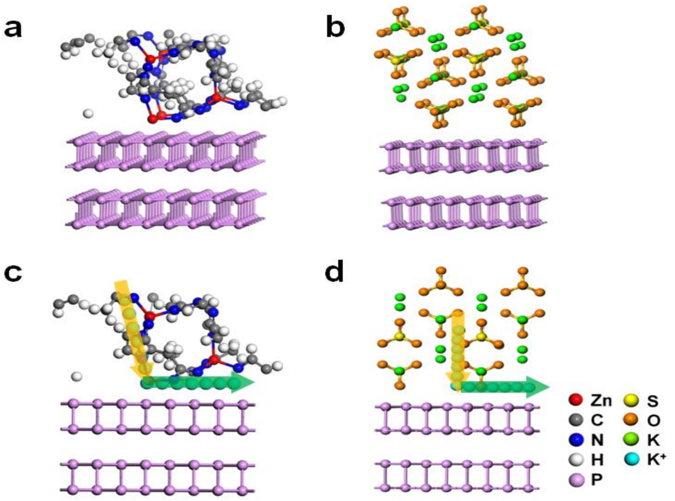

Fig. S26 (**a, b**) Two models used for theoretical calculations of (**a**) BP(020)-MOF(112), and (**b**) BP(020)-K_2SO_4(002). (**c, d**) The optimal migration pathways for the two systems of (**c**) BP(020)-MOF(112) and (**d**) BP(020)-K_2SO_4(002)

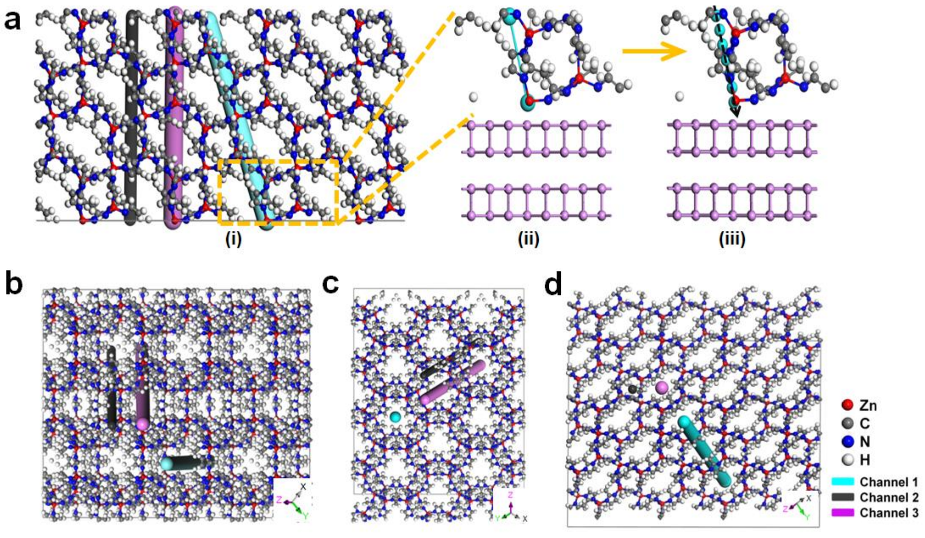

Fig. S27 (**a**) Procedure of identifying the migration pathway in the model of

BPNS@MOF when K ions across MOF. (**b-d**) Different pathways of K ions through MOF (the MOF is 2×2×2 supercell and cut along (112) plane with thickness of 27.74 Å). (**b**) top view. (**c**) view that perpendicular to channel 1. (**d**) view that perpendicular to channel 2 and 3.

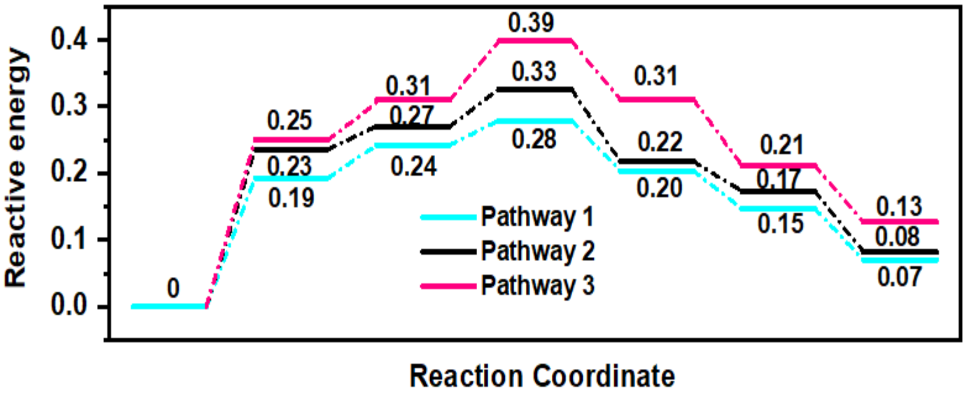

Fig. S28 Diffusion energy profile for three pathways when K ions pass through MOF in the model of BP(020)-MOF(112)

Note: The optimum pathway when K ions pass across MOF in BPNS@MOF is identified by comparing the energy barrier of several possible pathways. Fig. S27a shows the procedure of identifying the migration path in the model of the BPNS@MOF when K ions diffuse across MOF. Specifically, (i) Identify the possible channels inside MOF when K ions pass across the MOF layer, as shown in Fig. S27(b-d), there are three channels for K ion to pass through. (ii) Regard the fragment of MOF in the model of BP(020)-MOF(112) as an unit, and identify the start position and end position when K ions pass across the unit along the direction of the channel identified in (i). (iii) The two positions identified in (ii) are set as the start point and end point for K ions migration in the model of BP(020)-MOF(112). Three pathways of K ions across MOF in the model of BP(020)-MOF(112) are shown in Fig. 6a. Diffusion energy profiles of above three pathways for K ions diffusing through MOF are shown in Fig. S28, energy barriers are 0.28, 0.33, and 0.39 eV for pathway 1, 2, and 3, respectively. The results show that pathway 1 is more favorable for K ions to pass through in the model of BP(020)-MOF(112). Pathway 1 is chosen as the optimum pathway of K ions across MOF in BPNS@MOF compared with K ion across K_2SO_4 in BPNS/K_2SO_4 in Fig. 6c.

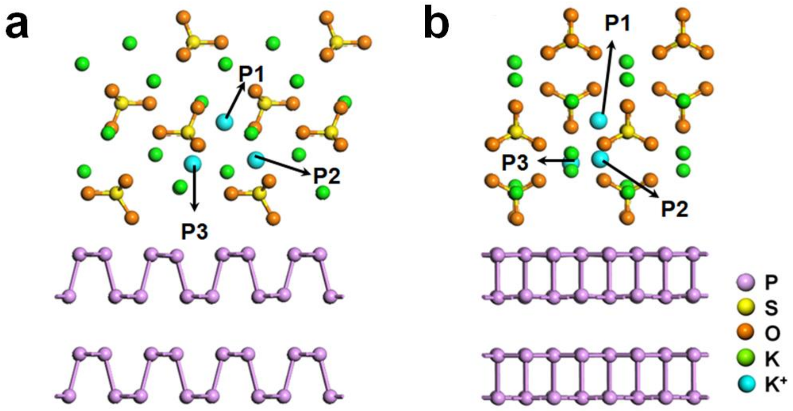

Fig. S29 Side views of different sites of K ions in K_2SO_4

Note: The optimum pathway when K ions across K_2SO_4 in BPNS/K_2SO_4 is identified by the energetically favorable adsorption site. The adsorption energy (E_{ads}) at different sites in the BPNS/ K_2SO_4 system is calculated by the following formula:

$$E_{ads} = E(BPNS/K_2SO_4{+}K) - E(BPNS/K_2SO_4) - E(K) \quad (S3)$$

where E(BPNS/K_2SO_4+K) is the total energy of K atom on the BPNS/K_2SO_4, E(BPNS/K_2SO_4) is the energy of the BPNS/K_2SO_4 and E(K) is the energy of a single K atom. On the basis of the definition, a larger negative value of E_{ads} suggests a stronger affinity of K atoms bonded with K_2SO_4 and thus a more stable system. As shown in Fig. S29, there are three positions in K_2SO_4, where P1 is in the middle of four S atoms, P2 is in the middle of four O atoms, and P3 is in the middle of four K atoms and is under one S atom. The adsorption energy is -0.62eV, -0.34eV and -0.28 eV for P1, P2, P3 in K_2SO_4, respectively. Thus, P1 is the energetically favorable adsorption site for K ions, and the optimum pathway when K ions pass across K_2SO_4 is along the direction of P1 and its equivalent site (Fig. S26d).

Table S1 Zinc atom density of different crystal planes in ZIF-8 MOF

Lattice plane	Number of zinc atom (n)	lattice parameter			Sectional area (nm^2)	Density of zinc atom (n/nm^2)
		a(Å)	b(Å)	θ(°)		
(011)	6	14.71	14.71	70.52	2.0400	2.94
(002)	6	16.99	16.99	90	2.8866	2.08
(112)	12	24.02	14.71	90	3.5333	3.40
(013)	12	28.17	16.99	72.45	4.5633	2.63
(222)	6	24.02	24.02	60	4.9966	1.20

Note: The calculation results based on a single MOF unit cell. The sectional area is calculated by the formula of $S = ab\sin\theta$, where a and b are two side length of the parallelogram cross section, θ is the acute angle between a and b.

Table S2 Specific capacity and capacity retention of the BPNS@MOF and BPNS electrodes at different current densities for KIBs

Current Density ($mA\ g^{-1}$)	Specific Capacity ($mAh\ g^{-1}$)		Capacity Retention (%)	
	BPNS@MOF	BPNS	BPNS@MOF	BPNS
50	301	216	/	/
100	222	148	73.7	68.7
200	192	123	86.4	83.2
500	162	84	84.5	68.0
1000	143	59	88.2	70.5
Back to 50 (compared with the 10th cycle)	261	155	97.2	77.6

Table S3 Fitting parameters of electrochemical impedance spectra for the BPNS electrode and the BPNS@MOF electrodes with different MOF contents for KIBs before cycling

Sample	R_s	CPE_1		R_f	CPE_2		R_{ct}	Zw		
	(Ω)	CPE_1-T	CPE_1-P	(Ω)	CPE_2-T	CPE_2-P	(Ω)	Zw-R	Zw-T	Zw-P
BPNS	9.3	2.2267E-3	0.5073	236.7	2.0235E-5	0.9845	1286	179.9	0.4374	0.4432
BPNS@MOF	15.9	5.5851E-5	0.7046	34.9	3.9199E-5	0.9286	750	9.1	0.0273	0.4415
BPNS@MOF-2	4.8	5.3546E-4	0.7602	3.0	3.1259E-5	0.9374	1203	46.9	0.0371	0.4516
BPNS@MOF-3	2.8	1.4481E-5	0.8813	91.2	2.9851E-5	0.8474	1980	304.3	0.5140	0.4395

Table S4 Comparison of performance of our work and the state-of-the-art KIC full cells

Electrode Materials	Current Density ($mA\ g^{-1}$)	Cycle number (n)	Retention (%)	Reference
CNSs//AC	2000	5000	80	2
NGC//KTO	1000	5000	75.5	3
PB//AC	2000	1200	98	4
CTP@C//AC	5000	4000	75.9	5
NPG//AC	1000	1000	66	6
K-V_2C//$K_xMnFe(CN)_6$	1000	200	95	7
K_2TP//AC	100	500	97.7	8
SC//AC	700	1500	97.5	9
N-$MoSe_2$/G//AC	1000	3000	75.2	10
U-Co_2P@rGO//AC	1000	1000	68	11
CNT@PC//APC	50	10000	89.6	12
S-N-PCN//AC	1000	3000	86.4	13
N-CNTs//LSG	400	5000	91.8	14
NHCS//ANHCS	2000	5000	80.4	15
$K_{0.45}Mn_{0.5}Co_{0.5}O_2$//AC	10000	30000	87.8	16
HC//AC	400	500	42	17
CFMS//CFMS	2000	10000	90	18
BPNS@MOF	1000	6500	99.5	This work

Supplementary References

[S1] R. Jain, P. Hundekar, T. Deng, X. Fan et al., Reversible alloying of phosphorene with potassium and its stabilization using reduced graphene oxide buffer layers. ACS Nano **13**, 14094–14106 (2019). https://doi.org/10.1021/acsnano.9b06680

[S2] J. Chen, B. Yang, H. Hou, H. Li et al., Disordered, large interlayer spacing, and oxygen-rich carbon nanosheets for potassium ion hybrid capacitor. Adv. Energy Mater. **9**, 1803894 (2019). https://doi.org/10.1002/aenm.201803894

[S3] S. Dong, Z. Li, Z. Xing, X. Wu, X. Ji et al., Novel potassium-ion hybrid capacitor based on an anode of $K_2Ti_6O_{13}$ microscaffold. ACS Appl. Mater. Interfaces **10**, 15542–15547 (2018). https://doi.org/10.1021/acsami.7b15314

[S4] L. Zhou, M. Zhang, Y. Wang, Y. Zhu et al., Cubic prussian blue crystals from a facile one-step synthesis as positive electrode material for superior potassium-ion capacitors. Electrochim. Acta **232**, 106–113 (2017). https://doi.org/10.1016/j.electacta.2017.02.096

[S5] Z. Zhang, M. Li, Y. Gao, Z. Wei, M. Zhang et al., Fast potassium storage in hierarchical $Ca_{0.5}Ti_2(PO_4)_3$@C microspheres enabling high-performance potassium-ion capacitors. Adv. Funct. Mater. **28**, 1802684 (2018). https://doi.org/10.1002/adfm.201802684

[S6] Y. Luan, R. Hu, Y. Fang, K. Zhu et al., Nitrogen and phosphorus dual-doped multilayer graphene as universal anode for full carbon-based lithium and potassium ion capacitors. Nano-Micro Lett. **11**, 1–13 (2019). https://doi.org/10.1007/s40820-019-0260-6

[S7] F. Ming, H. Liang, W. Zhang, J. Ming, Y. Lei et al., Porous MXenes enable high performance potassium ion capacitors. Nano Energy **62**, 853–860 (2019). https://doi.org/10.1016/j.nanoen.2019.06.013

[S8] Y. Luo, L. Liu, K. Lei, J. Shi, G. Xu et al., A nonaqueous potassium-ion hybrid capacitor enabled by two-dimensional diffusion pathways of dipotassium terephthalate. Chem. Sci. **10**, 2048–2052 (2019). https://doi.org/10.1039/C8SC04489A

[S9] L. Fan, K. Lin, J. Wang, R. Ma, B. Lu, A nonaqueous potassium-based battery-supercapacitor hybrid device. Adv. Mater. **30**, 18002 (2018). https://doi.org/10.1002/adma.201800804

[S10] Y. Yi, Z. Sun, C. Li, Z. Tian, C. Lu et al., Designing 3D biomorphic nitrogen-doped $MoSe_2$/graphene composites toward high-performance potassium-ion capacitors. Adv. Funct. Mater. **30**, 1903878 (2020). https://doi.org/10.1002/adfm.201903878

[S11] Y. Wang, Z. Zhang, G. Wang, X. Yang, Y. Sui et al., Ultrafine Co_2P nanorods wrapped by graphene enable a long cycle life performance for a hybrid

potassium-ion capacitor. Nanoscale Horiz. **4**, 1394–1401 (2019). https://doi.org/10.1039/C9NH00211A

[S12] G. Lu, H. Wang, Y. Zheng, H. Zhang, Y. Yang et al., Metal-organic framework derived N-doped CNT@porous carbon for high-performance sodium- and potassium- ion storage. Electrochim. Acta **319**, 541–551 (2019). https://doi.org/10.1016/j.electacta.2019.07.026

[S13] X. Hu, Y. Liu, J. Chen, L. Yi, H. Zhan et al., Fast redox kinetics in bi-heteroatom doped 3D porous carbon nanosheets for high-performance hybrid potassium-ion battery capacitors. Adv. Energy Mater. **9**, 1901533 (2019). https://doi.org/10.1002/aenm.201901533

[S14] M. Moussa, S.A. Al-Bataineh, D. Losic, D.P. Dubal, Engineering of high-performance potassium-ion capacitors using polyaniline-derived N-doped carbon nanotubes anode and laser scribed graphene oxide cathode. Appl. Mater. Today **16**, 425–434 (2019). https://doi.org/10.1016/j.apmt.2019.07.003

[S15] D. Qiu, J. Guan, M. Li, C. Kang, J. Wei et al., Kinetics enhanced nitrogen-doped hierarchical porous hollow carbon spheres boosting advanced potassium-ion hybrid capacitors. Adv. Funct. Mater. **29**, 1903496 (2019). https://doi.org/10.1002/adfm.201903496

[S16] H.V. Ramasamy, B. Senthilkumar, P. Barpanda, Y.S. Lee, Superior potassium-ion hybrid capacitor based on novel P_3-type layered $K_{0.45}Mn_{0.5}Co_{0.5}O_2$ as high capacity cathode. Chem. Eng. J. **368**, 235–243 (2019). https://doi.org/10.1016/j.cej.2019.02.172

[S17] Z. Xu, M. Wu, Z. Chen, C. Chen, J. Yang et al., Direct structure-performance comparison of all-carbon potassium and sodium ion capacitors. Adv. Sci. **6**, 1802272 (2019). https://doi.org/10.1002/advs.201802272

[S18] Y. Feng, S. Chen, J. Wang, B. Lu, Carbon foam with microporous structure for high performance symmetric potassium dual-ion capacitor. J. Energy Chem. **43**, 129–138 (2020). https://doi.org/ 10.1016/j.jechem.2019.08.013

[S19] W. Zhang, W. Pang, V. Sencadas, Z. Guo, Understanding high-energy-density Sn_4P_3 anodes for potassium-ion batteries. Joule **2**, 1534–1547 (2018). https://doi.org/10.1016/j.joule.2018.04.022

[S20] L. Deng, Y. Zhang, R. Wang, M. Feng, X. Niu et al., Influence of KPF_6 and KFSI on the performance of anode materials for potassium ion batteries: A case study of MoS_2. ACS Appl. Mater. Interfaces **11**, 22449–22456 (2018). https://doi.org/10.1021/acsami.9b06156

[S21] X. Ren, M. He, N. Xiao, W.D. McCulloch, Y. Wu, Greatly enhanced anode stability in K-oxygen batteries with an in situ formed solvent and oxygen-impermeable protection layer. Adv. Energy Mater. **7**, 1601080 (2016). https://doi.org/10.1002/aenm.201601080

[S22] Q. Zhang, J. Mao, W. Pang, T. Zheng, V. Sencadas et al., Boosting the potassium storage performance of alloy-based anode materials via electrolyte salt chemistry. Adv. Energy Mater. **8**, 1703288 (2018). https://doi.org/10.1002/aenm.201703288

[S23] N. Xiao, W.D. McCulloch, Y. Wu, Reversible dendrite-free potassium plating and stripping electrochemistry for potassium secondary batteries. J. Am. Chem. Soc. **139**, 9475–9478 (2017). https://doi.org/10.1021/jacs.7b04945

[S24] H. Wang, D. Yu, X. Wang, Z. Niu, M. Chen, L. Cheng et al., Electrolyte chemistry enables simultaneous stabilization of potassium metal and alloying anode for potassium-ion batteries. Angew. Chem. Int. Ed. **58**, 16451–16455 (2019). https://doi.org/10.1002/anie.201908607

[S25] H. Zhong, J. Wang, Y. Zhang, W. Xu, W. Xing et al., ZIF-8 derived graphene-based nitrogen-doped porous carbon sheets as highly efficient and durable oxygen reduction electrocatalysts. Angew. Chem. Int. Ed. **53**, 14235–14239 (2014). https://doi.org/10.1002/ange.201408990

[S26] U. P. N. Tran, K. K. A. Le, N. T. S. Phan, Expanding applications of metal-organic frameworks: zeolite imidazolate framework ZIF-8 as an efficient heterogeneous catalyst for the knoevenagel reaction. ACS Catal. **1**, 120–127 (2011). https://doi.org/10.1021/cs1000625

[S27] Y. Hu, H. Kazemian, S. Rohani, Y. Huang, Y. Song, In situ high pressure study of ZIF-8 by FTIR spectroscopy. Chem. Commun. **47**, 12694–12696 (2011). https://doi.org/10.1039/C1CC15525C

[S28] X. Lou, Y. Ning, C. Li, X. Hu, M. Shen, B. Hu, Bimetallic zeolite imidazolate framework for enhanced lithium storage boosted by the redox participation of nitrogen atoms. Sci. China Mater. **61**, 1040–1048 (2018). https://doi.org/10.1007/s40843-017-9200-5

[S29] Z. Shen, L. Cao, C.D. Rahu, C. Wang, Least squares galvanostatic intermittent titration technique (LS-GITT) for accurate solid phase diffusivity measurement. J. Electrochem. Soc. **160**, A1842–A1846 (2013). https://doi.org/10.1149/2.084310jes

[S30] P. Wang, H. Yao, X. Liu, Y. Yin et al., Na^+/vacancy disordering promises high-rate Na-ion batteries. Sci. Adv. **4**, eaar6018 (2018). https://doi.org/10.1126/sciadv.aar6018

[S31] M. Huang, B. Xi, Z. Feng, F. Wu, D. Wei et al., New insights into the electrochemistry superiority of liquid Na–K Alloy in metal batteries. Small **15**, 1804916 (2019). https://doi.org/10.1002/ smll. 201804916

[S32] F. Ming, H. Liang, W. Zhang, J. Ming, Y. Lei et al., Porous MXenes enable high performance potassium ion capacitors. Nano Energy **62**, 853–860 (2019). https://doi.org/10.1016/j.nanoen. 2019.06.013
